# NOG1 downregulates type I interferon production by targeting phosphorylated interferon regulatory factor 3

**DOI:** 10.1371/journal.ppat.1011511

**Published:** 2023-07-06

**Authors:** Qiao Xue, Zixiang Zhu, Zhaoning Xue, Fan Yang, Weijun Cao, Xiangtao Liu, Huisheng Liu, Haixue Zheng

**Affiliations:** State Key Laboratory for Animal Disease Control and Prevention, College of Veterinary Medicine, Lanzhou University, Lanzhou Veterinary Research Institute, Chinese Academy of Agricultural Sciences, Lanzhou, China; McMaster University, CANADA

## Abstract

The innate immune system is the first line of the host’s defense, and studying the mechanisms of the negative regulation of interferon (IFN) signaling is important for maintaining the balance of innate immune responses. Here, we found that the host GTP-binding protein 4 (NOG1) is a negative regulator of innate immune responses. Overexpression of NOG1 inhibited viral RNA- and DNA-mediated signaling pathways, and NOG1 deficiency promoted the antiviral innate immune response, resulting in the ability of NOG1 to promote viral replication. Vesicular stomatitis virus (VSV) and herpes simplex virus type 1 (HSV-1) infection induced a higher level of IFN-β protein in NOG1 deficient mice. Meanwhile, NOG1-deficient mice were more resistant to VSV and HSV-1 infection. NOG1 inhibited type I IFN production by targeting IRF3. NOG1 was also found to interact with phosphorylated IFN regulatory factor 3 (IRF3) to impair its DNA binding activity, thereby downregulating the transcription of IFN-β and downstream IFN-stimulated genes (ISGs). The GTP binding domain of NOG1 is responsible for this process. In conclusion, our study reveals an underlying mechanism of how NOG1 negatively regulates IFN-β by targeting IRF3, which uncovers a novel role of NOG1 in host innate immunity.

## Author summary

Although type I IFN benefits antiviral activity, IFN aberrant secretion can trigger diseases. The study of the mechanisms of innate immune response could contribute to better disease control or vaccine design. In the present study, we, for the first time, demonstrate that the host GTP-binding protein 4 (NOG1) acts as a repressor of host innate immune responses. NOG1 negatively regulates viral RNA- and DNA-mediated signaling pathways by inhibiting the DNA binding ability of IFN regulatory factor 3 (IRF3). Our study reveals a common mechanism of NOG1 in regulating antiviral innate immunity and broadens the regulation mechanisms targeting IRF3.

## Introduction

The innate immune system is the first line of the host’s defense that protects against invading pathogens. Host pattern recognition receptors (PRRs), including RIG-like receptors (RIG-I, MDA5, and LGP2), cyclic GMP-AMP synthase (cGAS), and NOD-like receptors (NOD2), play a critical role in innate immunity where they interact with pathogen-associated molecular patterns (PAMPs) to induce interferon (IFN) production which exerts antiviral functions [1]. To counteract host antiviral responses and maintain viral replication, viruses must overcome host innate immune responses to establish a productive infection. After viral RNA recognition, RIG-I recruits the adaptor molecule mitochondrial antiviral signaling protein (MAVS) to activate the TANK-binding kinase 1 (TBK1) and the inhibitor of ĸB kinase α/β (IKK α/β) complex, resulting in the activation of interferon regulatory factor 3/7 (IRF3/7) and nuclear factor-ĸB (NF-ĸB), which induces the production of type I interferon (IFN-I, IFN-α, and IFN-β) [2]. After viral DNA recognition, cGAS recruits the stimulator of interferon genes (STING), which further activates TBK1 and IRF3 to induce IFN-I production [3]. Then, the secreted IFN-I binds to receptors on the cell surface and activates the kinase/signal transducer and activator of transcription (JAK/STAT), resulting in the phosphorylation of STAT1/2. The phosphorylated STAT1/2 interacts with IRF9 to form an IFN-stimulated gene factor 3 (ISGF3) complex, which binds to the IFN-stimulated response element (ISRE) to activate the transcription of IFN-stimulated genes (ISGs), leading to cellular antiviral state [4,5].

Many cellular factors can regulate the IFN-I-inducing pathway. IRF3 is a major transcription factor that regulates IFN-I production [6–8]. Upon viral infection, cytoplasmic IRF3 is phosphorylated and forms dimers. Subsequently, the activated IRF3 enters the nucleus and associates with CREB-binding protein (CBP)/p300 coactivators to form a complex and binds to the promoter of the targeted genes, resulting in the transcription of IFN-I and the downstream ISGs [9]. Although IFN is beneficial for antiviral activity, IFN aberrant secretion can trigger inflammatory disorders or other diseases [10,11]. Regulation of IRF3 includes positive and negative mechanisms. The positive mechanisms comprise increased expression and activation of IRF3, and the negative ones contain decreased expression and inhibition of IRF3 activity [12]. Some negative regulators have been identified to impair IRF3 functions. For instance, peptidylprolyl cis-trans isomerase NIMA-interacting 1 (Pin 1) degrades IRF3 through the proteasome pathway, thereby inhibiting the innate immune responses [13], RUN domain Beclin-1-interacting cysteine-rich domain containing (Rubicon) interacts with IRF3 and inhibits its dimerization [14], and the cell growth-regulating nucleolar protein LYAR suppresses IFN production by targeting phosphorylated IRF3 [15]. The negative regulation of IRF3-mediated IFN signaling is important for maintaining the balance of innate immune responses.

GTP-binding protein 4 (NOG1, also known as GTPBP4, NGB, or CRFG) is conserved across eukaryotes from yeast to humans and is a novel member of GTPases belonging to the guanine nucleotide-binding proteins family [16]. NOG1 locates in the nucleolus, which is involved in the biogenesis of the 60 s ribosomal subunit, and the GTP binding motif is required for function [17]. NOG1 is a multi-functional protein and is also involved in the cell cycle, DNA mismatch repair system, PKM2-dependent glucose metabolism, and cancer [18–21]. However, the impact of NOG1 on innate immune responses still needs to be investigated.

This study aimed to examine how NOG1 regulates the innate immune response. We found that NOG1 negatively regulated RNA and DNA virus-induced innate immune response, promoting viral replication *in vitro and in vivo*. Further studies found that NOG1 suppressed IFN production by inhibiting the binding of IRF3 to the promoter. Our findings uncovered a novel role of the NOG1 protein as a negative regulator of innate immune responses.

## Results

### NOG1 negatively regulates viral RNA- and DNA-triggered signaling

**Fig 1:**
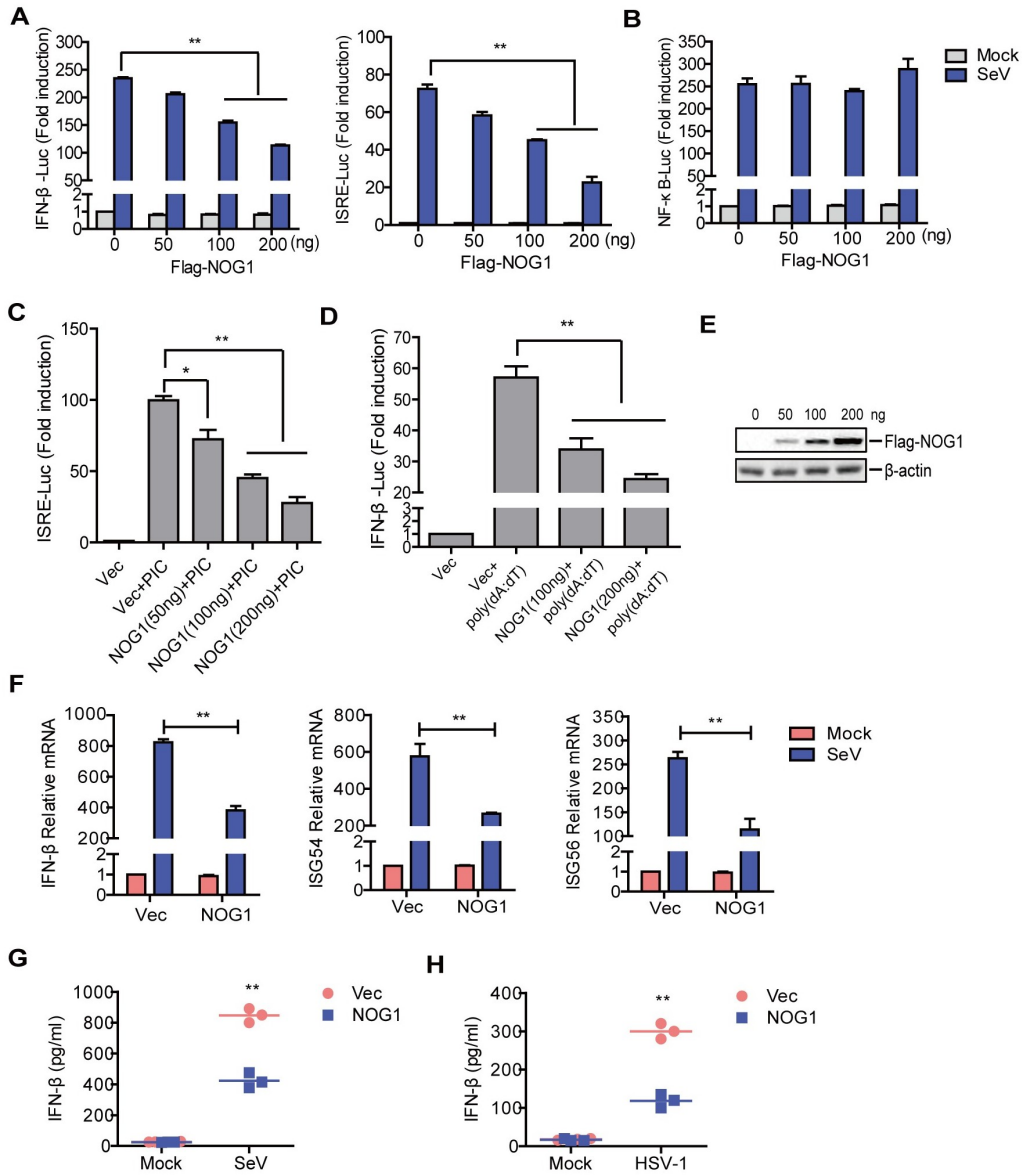
NOG1 negatively regulates viral RNA- and DNA-triggered signaling. (A-E) HEK-293T cells were transfected with 0.1 μg of IFN-β-Luc, ISRE-Luc, or NF-ĸB-Luc, and 0.01 μg of pRL-TK plasmid along with increasing Flag-NOG1-expressing plasmids. At 24 hpt, the cells were infected with SeV (A and B) or transfected with poly(I:C) (PIC) (C). The promoter activation of IFN-β, ISRE, and NF-ĸB was determined by the dual-specific luciferase assay kit. (D) HEK-293 cells were transfected with 0.1 μg of IFN-β-Luc and 0.01 μg of pRL-TK plasmid along with Flag-NOG1-expressing plasmids. At 24 hpt, the cells were transfected with poly(dA:dT). The IFN-β promoter activity was determined by the dual-specific luciferase assay kit. (E) The expression of increasing NOG1 was detected by Western blotting. (F) HEK-293T cells were transfected with 1 μg of Flag empty vector or Flag-NOG1 expression plasmid. At 24 hpt, the cells were infected with SeV for an additional 12 h. The mRNA expression of IFN-β, ISG54, and ISG56 was measured by qPCR. (G-H) HEK-293T cells (G) or HeLa cells (H) were transfected with 1 μg of Flag empty vector or Flag-NOG1 expression plasmid. At 24 hpt, the cells were infected with SeV and HSV-1, respectively. The expression of IFN-β protein in the supernatant was detected by ELISA kit.

To identify candidate molecules involved in innate immune response, we screened ~20 independent proteins for their abilities to regulate IFN-β activity using luciferase reporter assays and identified NOG1 as a candidate protein. As shown in Fig 1A and 1B, overexpression of NOG1 significantly inhibited Sendai virus (SeV)-triggered activation of IFN-β promoter (which is driven by ISRE and ĸB enhancers) and ISRE (interferon-stimulated response element) promoter in a dose-dependent manner. However, overexpression of NOG1 did not affect NF-ĸB-Luc activity.

To further confirm the impact of NOG1 on innate immune response, HEK-293T cells were transfected with ISRE-Luc along with Flag-NOG1 expression plasmids and poly(I:C) (a mimic of viral dsRNA). The ISRE promoter activity was detected using a Dual-Luciferase assay kit. The results showed that overexpression of NOG1 inhibited poly(I:C)-induced ISRE promoter activity in a dose-dependent manner (Fig 1C). HEK-293T cells expressing the SV40 large T antigen lack endogenous STING, while HEK-293 cells without SV40 large T antigen express high levels of STING and can induce interferon antiviral responses to cytosolic DNA [22–24]. To investigate the impact of NOG1 on innate immune response after DNA stimulation, HEK-293 cells were transfected with IFN-β promoter along with Flag-NOG1 expression plasmids and poly(dA:dT) (one strand of double-stranded DNA). The IFN-β promoter activity was detected. Overexpression of NOG1 also inhibited poly(dA:dT)-induced IFN-β promoter activity (Fig 1D). The expression of increasing NOG1 was detected by Western blotting (Fig 1E).

To investigate whether the NOG1 protein affected the expression of IFN-β and IFN-stimulated genes (ISGs), the mRNA expression of IFN-β, ISG54, and ISG56 in HEK-293T cells that were transfected with Flag-NOG1 expression plasmids and infected with SeV was measured. NOG1 significantly inhibited SeV-induced IFN-β, ISG54, and ISG56 mRNA expression (Fig 1F). We further measured the effect of NOG1 on IFN-β protein expression in SeV-infected HEK-293T cells and herpes simplex virus type 1 (HSV-1)-infected HeLa cells, which showed that NOG1 significantly inhibited SeV- and HSV-1-induced IFN-β protein secretion (Fig 1G and 1H). These results indicated that NOG1 is an important inhibitor of viral RNA- and DNA-mediated signaling pathways.

### NOG1 deficiency promotes the antiviral innate immune response

**Fig 2:**
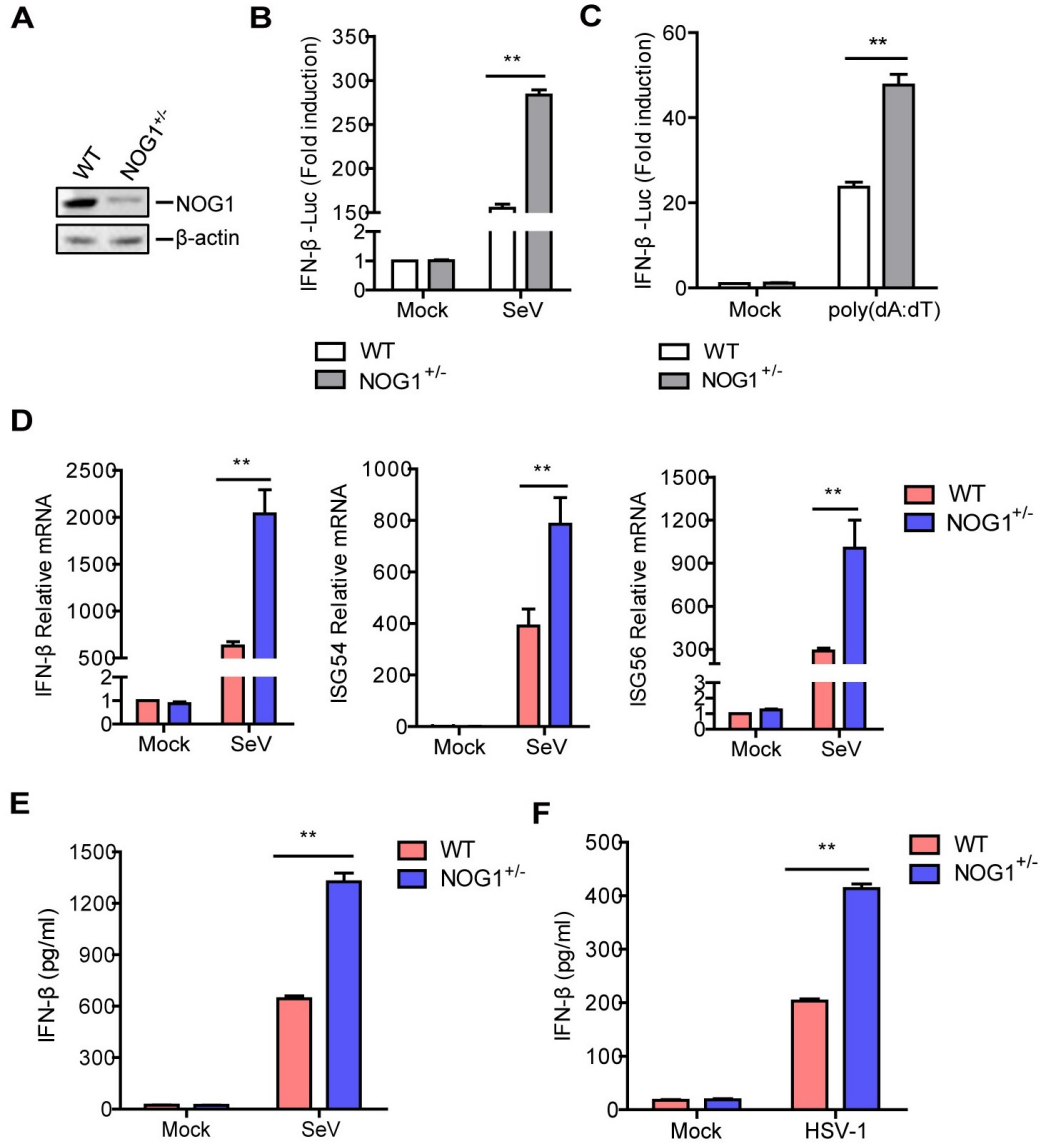
NOG1 deficiency promotes the antiviral innate immune response. (A) The expression of NOG1 in the WT and NOG1^+/-^ cells was detected by Western blotting. (B-C) WT and NOG1^+/-^ cells were transfected with 0.1 μg of IFN-β-Luc and 0.01 μg of the pRL-TK plasmid. At 24 hpt, the cells were infected with SeV (HEK-293T cells, B) or transfected with poly(dA:dT) (HEK-293 cells, C). The promoter activation of IFN-β was determined by the dual-specific luciferase assay kit. (D) WT and NOG1^+/-^ HEK-293T cells were infected with SeV for 12 h. The mRNA expression of IFN-β, ISG54, and ISG56 was detected by qPCR. (E-F) WT and NOG1^+/-^ HEK-293T cells (E) or HeLa cells (F) were infected with SeV and HSV-1, respectively. The expression of IFN-β protein in the supernatant was detected by ELISA kit.

We next determined whether endogenous NOG1 is required for viral RNA- and DNA-triggered signaling. We determined that cells lacking NOG1 could not survive using the CRISPR/Cas9 system in the knockout cell experiments. Therefore, NOG1 heterozygous knockout (NOG1^+/-^) cells were used in subsequent experiments. The expression of NOG1 was decreased in the NOG1^+/-^ cells compared to that in the WT cells (Fig 2A). We evaluated cell viability and host proteins synthesis in the WT and NOG1^+/-^ cells using CCK-8 solution and puromycin, respectively. The heterozygous knockout of NOG1 did not affect cell viability and host proteins synthesis compared to the WT cells (S1A and S1B Fig).

WT and NOG1^+/-^ cells were transfected with IFN-β-Luc and pRL-TK plasmids along with SeV (HEK-293T cells) or poly(dA:dT) (HEK-293 cells), the IFN-β promoter activity was detected by Dual-Luciferase assay kit. The results showed that SeV- and poly(dA:dT)-induced IFN-β promoter activity was significantly enhanced in the NOG1^+/-^ cells than in the WT cells (Fig 2B and 2C).

The expression of IFN-β, ISG54, ISG56, and IL-1β in the WT and NOG1^+/-^ cells infected with SeV was also evaluated. As expected, NOG1 deficiency significantly promoted SeV-induced IFN-β, ISG54, and ISG56 mRNA expression (Fig 2D). However, NOG1 deficiency did not affect IL-1β mRNA expression (S2A Fig). IL-1β is a key mediator of the inflammatory response [25]. Therefore, we further evaluated the effect of NOG1 on IL-1β expression. THP1 cells transfected with Flag-NOG1 expression plasmids were mock-infected or infected with SeV, and the expression of pro-IL-1β and IL-1β was determined. The results showed that overexpression of NOG1 did not affect SeV-induced IL-1β protein expression (S2B Fig), which indicated that NOG1 did not regulate the production of inflammatory cytokines.

NOG1 deficiency also facilitated IFN-β protein secretion in SeV-infected HEK-293T cells and HSV-1-infected HeLa cells (Fig 2E and 2F). In addition, the impact of NOG1 on IFN-β protein secretion was also assessed in THP1 and A549 cells. NOG1 siRNA was used to knockdown NOG1 expression in THP1 and A549 cells. Negative control (NC) siRNA was used as a negative control. THP1 and A549 cells transfected with NOG1 or NC siRNA were infected with HSV-1 or VSV. The levels of IFN-β protein were determined by ELISA. Again, NOG1 deficiency facilitated HSV-1-induced IFN-β protein secretion in THP1 cells and VSV-induced IFN-β protein secretion in THP1 and A549 cells (S3 Fig). These results indicated that NOG1 deficiency promoted viral RNA- and DNA-mediated innate immune responses.

### NOG1 promotes viral replication *in vitro*

**Fig 3:**
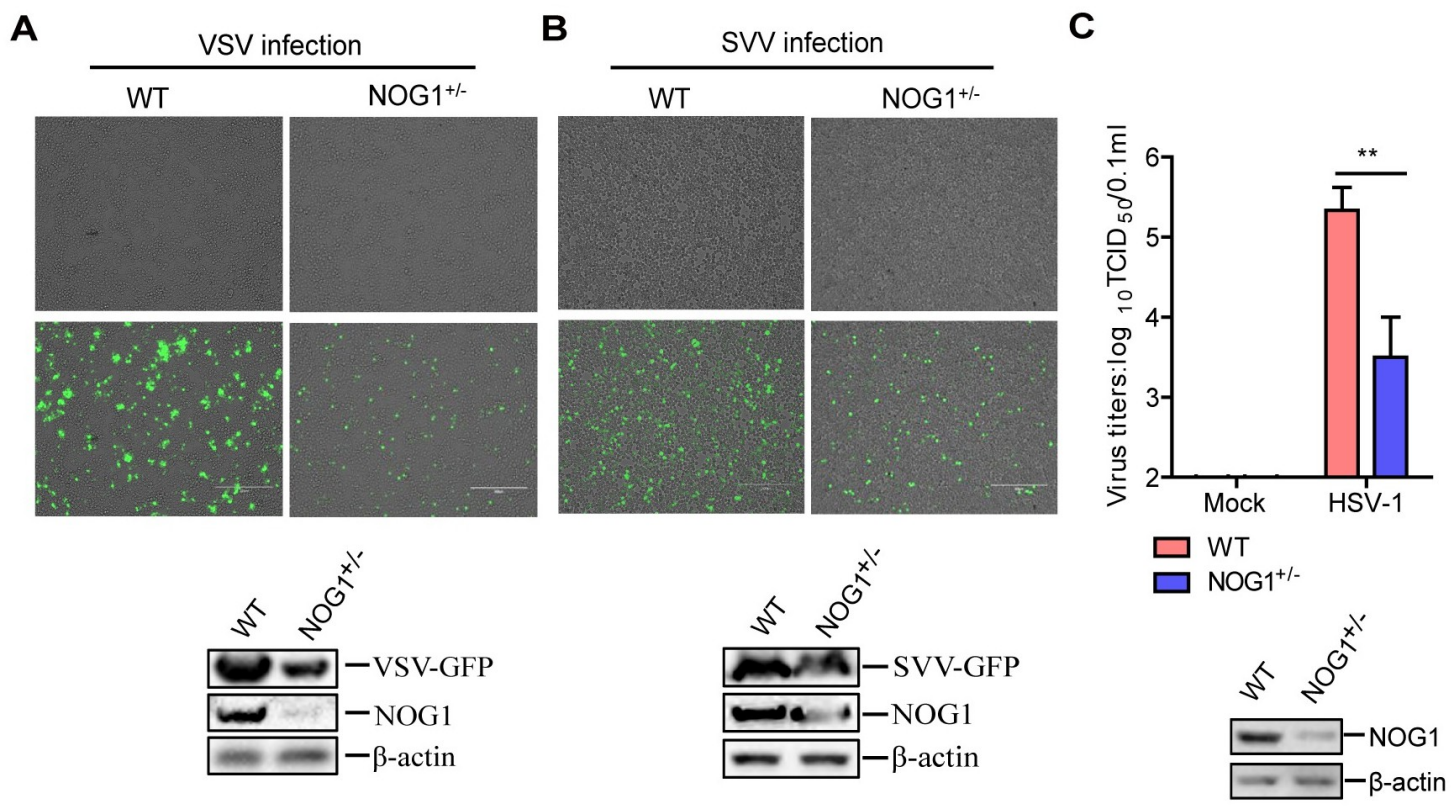
NOG1 promotes viral replication in cells. (A-B) WT and NOG1^+/-^ HeLa cells (A) and HEK-293T cells (B) were infected with VSV-GFP (1 MOI) and SVV-GFP (1 MOI), respectively. The fluorescence images were visualized using a Nikon Eclipse 80i fluorescence microscope. The protein expression of GFP was detected by Western blotting. (C) WT and NOG1^+/-^ HeLa cells were infected with HSV-1 (1 MOI) for 24 h. The HSV-1 titers were determined by TCID_50_ assay.

Type I IFN plays a critical antiviral effect during viral infection. Accordingly, whether NOG1 had a regulatory role in viral replication was further explored. HeLa and HEK-293T cells were mock-infected or infected with GFP-tagged vesicular stomatitis virus (VSV) or Seneca Valley virus (SVV), respectively. The fluorescence and protein expression of GFP was detected and compared. As shown in Fig 3A, there were more VSV GFP-positive populations in the WT cells than in the NOG1^+/-^ cells, and the expression of GFP decreased in the NOG1^+/-^ cells compared to WT cells. Similar to VSV, the levels of SVV GFP were also reduced in the NOG1^+/-^ cells compared to that in the WT cells (Fig 3B).

The regulatory effect of NOG1 on HSV-1 replication was also investigated. We assessed HSV-1 yields in the WT and NOG1^+/-^ HeLa cells. The viral titers were significantly lower in the NOG1^+/-^ cells than in the WT cells during HSV-1 infection, and the deficiency of NOG1 protein in the NOG1^+/-^ cells was confirmed by Western blotting (Fig 3C). These results indicated that NOG1 promotes both RNA virus and DNA virus replication *in vitro*.

To demonstrate that the inhibition of viral replication is indeed due to changes in IFN-β levels, WT and NOG1^+/-^ HeLa cells were incubated with or without an increasing amount of anti-IFN-β antibody, as described previously [26]. VSV titers were significantly enhanced in the WT and NOG1^+/-^ cells treated with anti-IFN-β antibody compared to that in the cells without anti-IFN-β antibody treatment, and there was no significant difference in viral titers between WT and NOG1^+/-^ cells treated with anti-IFN-β antibody (S4A Fig). The results suggested that NOG1 indeed promoted VSV replication depending on IFN-β.

Previous results have shown that TBK1-mediated signal transduction was abnormal in IBRS-2 cells, and the innate immune response-related pathways were not activated in IBRS-2 cells during RNA virus infection [27,28]. Therefore, IBRS-2 cells were selected to further evaluate the impact of NOG1 on SVV replication. IBRS-2 cells transfected with Flag-NOG1 expression plasmids were infected with SVV. No significant difference in viral titers was observed (S4B Fig), suggesting that NOG1 promoted SVV replication depending on IFN-β. These results indicated that NOG1 regulates viral replication depending on the expression of IFN-β.

### NOG1 deficient mice are more resistant to VSV and HSV-1 infection

**Fig 4:**
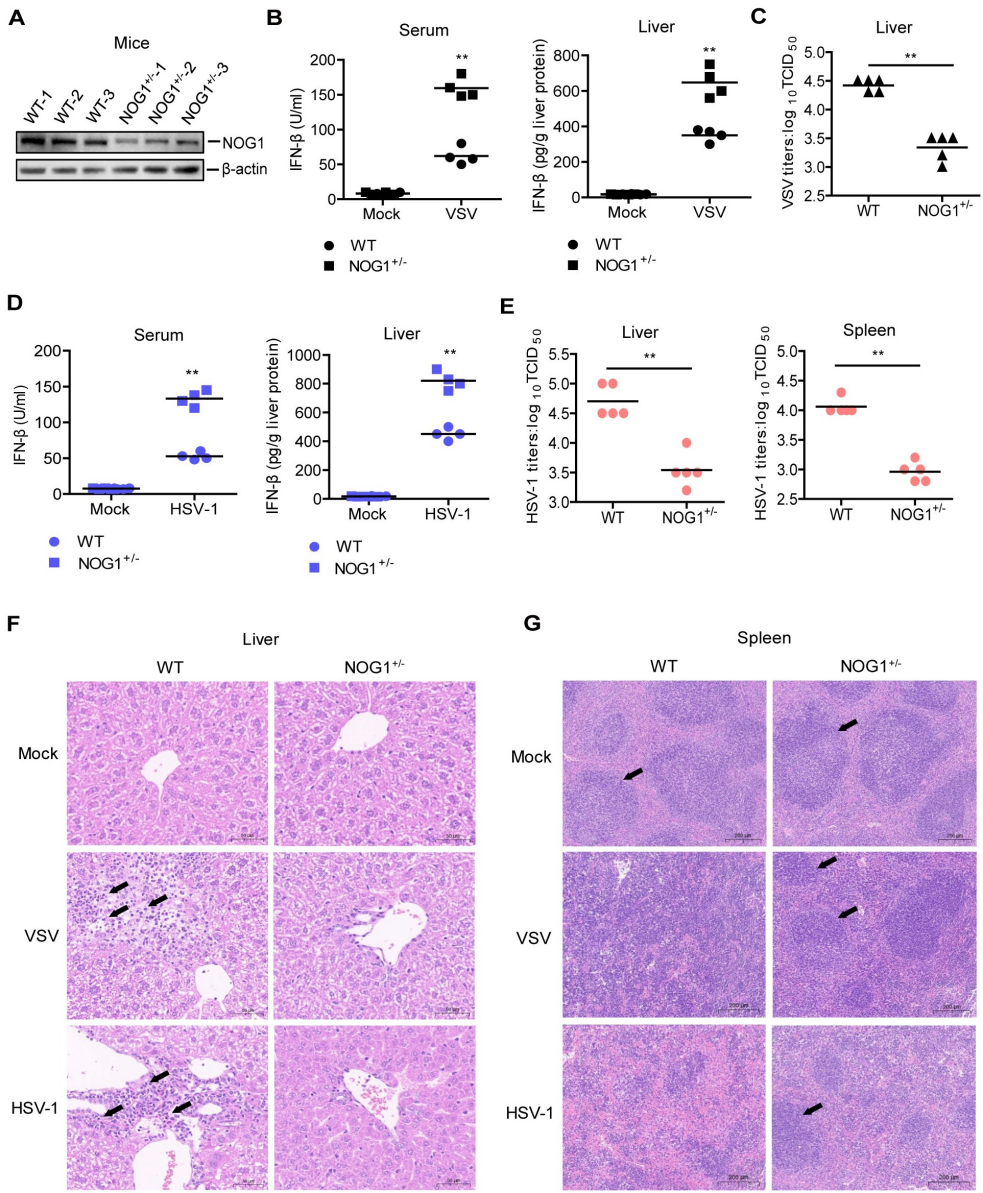
NOG1 deficient mice are more resistant to VSV and HSV-1 infection. (A) The expression of NOG1 in the liver of WT and NOG1^+/-^ mice was detected by Western blotting. (B-C) WT and NOG1^+/-^ mice were intraperitoneally injected with VSV (6×10^7^ PFU) for 48 h. The expression of IFN-β in the serum and liver of mice (n = 4) was detected by ELISA kit (B), and viral titers in the liver of mice (n = 5) were detected by TCID_50_ assay (C). (D-E) WT and NOG1^+/-^ mice were intraperitoneally injected with HSV-1 (5×10^6^ PFU) for 48 h. The expression of IFN-β in the serum and liver of mice (n = 4) was detected by ELISA kit (D), and viral titers in the liver and spleen of mice (n = 5) were detected by TCID_50_ assay (E). (F-G) WT and NOG1^+/-^ mice were intraperitoneally injected with VSV (6×10^7^ PFU) or HSV-1 (5×10^6^ PFU) for 5 d. H&E staining was performed for histological examination of the liver and spleen of mice. Inflammatory cells in the liver and white pulp in the spleen are indicated by a black arrowhead.

To assess the physiologic relevance of NOG1 function, we evaluated the importance of NOG1 in antiviral host defense in mice. We determined that the NOG1^-/-^ mice are embryonic lethal in the knockout mice experiments. Therefore, the NOG1 heterozygous (NOG1^+/-^) mice were used in subsequent experiments. The reduction of NOG1 protein in the liver of NOG1^+/-^ mice was confirmed by Western blotting (Fig 4A). NOG1^+/-^ mice were born at the Mendelian ratio and all the mice developed normally and gained weight, similar to WT mice, which indicated that heterozygous knockout of NOG1 gene is dispensable for mouse development.

WT and NOG1^+/-^ mice were intraperitoneally injected with VSV or HSV-1. IFN-β expression and viral titers in the tissue of mice were monitored. The levels of IFN-β protein were significantly enhanced in the serum and liver of NOG1^+/-^ mice infected with VSV compared to that in the VSV-infected WT mice (Fig 4B). Furthermore, the VSV titers were significantly decreased in the liver of NOG1^+/-^ mice infected with VSV compared to that in the VSV-infected WT mice (Fig 4C). In addition, the viral load of VSV in the liver was further determined by immunohistochemical analysis. The positive stains were found in the VSV-infected mice, and the rate of positive cells was significantly reduced in the NOG1^+/-^ mice compared to that in the WT mice (S5A Fig), confirming that NOG1 deficient mice are more resistant to VSV infection. The impact of NOG1 on VSV-induced mice mortality was evaluated as well. The WT mice infected with VSV survived 50%, while the NOG1^+/-^ mice infected with VSV survived 83.3% at 15 dpi, suggesting that NOG1 deficiency resulted in lower mortality of the mice infected with VSV (S5B Fig).

Similar to VSV, HSV-1 infection induced a higher level of IFN-β protein and HSV-1 titers were significantly reduced in the NOG1^+/-^ mice compared to that in the WT mice (Fig 4D and 4E). These results indicated that NOG1 deficiency protected mice against VSV and HSV-1 infection.

To confirm whether NOG1 deficiency also reduced tissue injury after VSV and HSV-1 infection, the histological changes in the liver and spleen of WT and NOG1^+/-^ mice were detected and compared. There was no histological change in the liver and spleen of mock-infected WT and NOG1^+/-^ mice. Pathology examination showed that VSV and HSV-1 infection induced infiltration of inflammatory cells in the liver, and decreased infiltration of inflammatory cells was observed in the liver of NOG1^+/-^ mice compared to WT mice (Fig 4F). In addition, VSV and HSV-1 infection reduced the amount of the white pulp in the spleen and destroyed its structure, and less tissue damage morphology was observed in the spleen of NOG1^+/-^ mice compared to WT mice (Fig 4G). These results indicated that NOG1 deficiency protected mice against tissue injury during VSV and HSV-1 infection.

### IRF3 might be the potential target of the NOG1 protein

**Fig 5:**
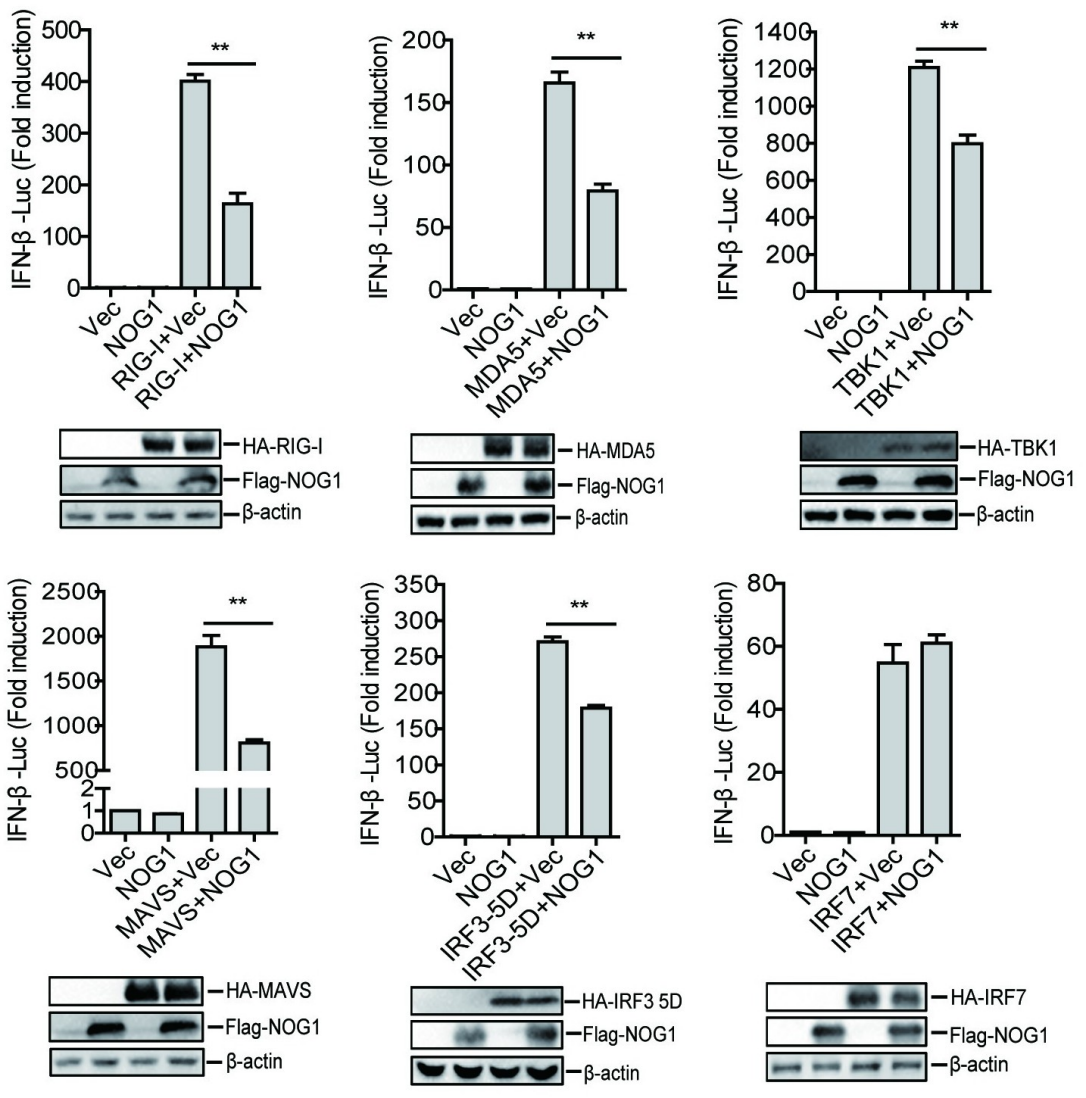
NOG1 inhibited IFN-β production by targeting IRF3. HEK-293T cells were transfected with 0.1 μg/well of IFN-β-Luc, 0.01 μg/well of pRL-TK plasmid along with 0.1 μg/well of Flag vector or Flag-NOG1 expressing plasmid, and HA-tagged RIG-I, MDA5, TBK1, MAVS, IRF3-5D, and IRF7 expressing plasmids. At 24 hpt, the activation of the IFN-β promoter was evaluated by the dual-specific luciferase assay kit.

The observed inhibition of type I IFN production by the NOG1 protein raises the possibility that NOG1 targets one or several components of the innate immune signaling pathway. To identify the potential target regulated by NOG1, HEK-293T cells were co-transfected with Flag-NOG1 expression plasmid and plasmids expressing each component of the innate immune signaling pathway (including RIG-I, MDA5, MAVS, TBK1, IRF3-5D, and IRF7), together with IFN-β-Luc and pRL-TK plasmids. The activity of the IFN-β promoter was determined by a Dual-Luciferase assay kit. The results showed that overexpression of these component molecules activated IFN-β promoter activity, while overexpression of NOG1 protein significantly inhibited the activation of the IFN-β promoter induced by all of these molecules, except for IRF7 (Fig 5). The results of Western blotting indicated that NOG1 protein did not affect the protein expression of these components (Fig 5). Meanwhile, our data indicated that NOG1 did not interact with RIG-I, MDA5, MAVS, and TBK1 (S6 Fig). Therefore, we speculated that the NOG1 protein targeted IRF3 to block type I IFN production.

### NOG1 interacts with phosphorylated IRF3

**Fig 6:**
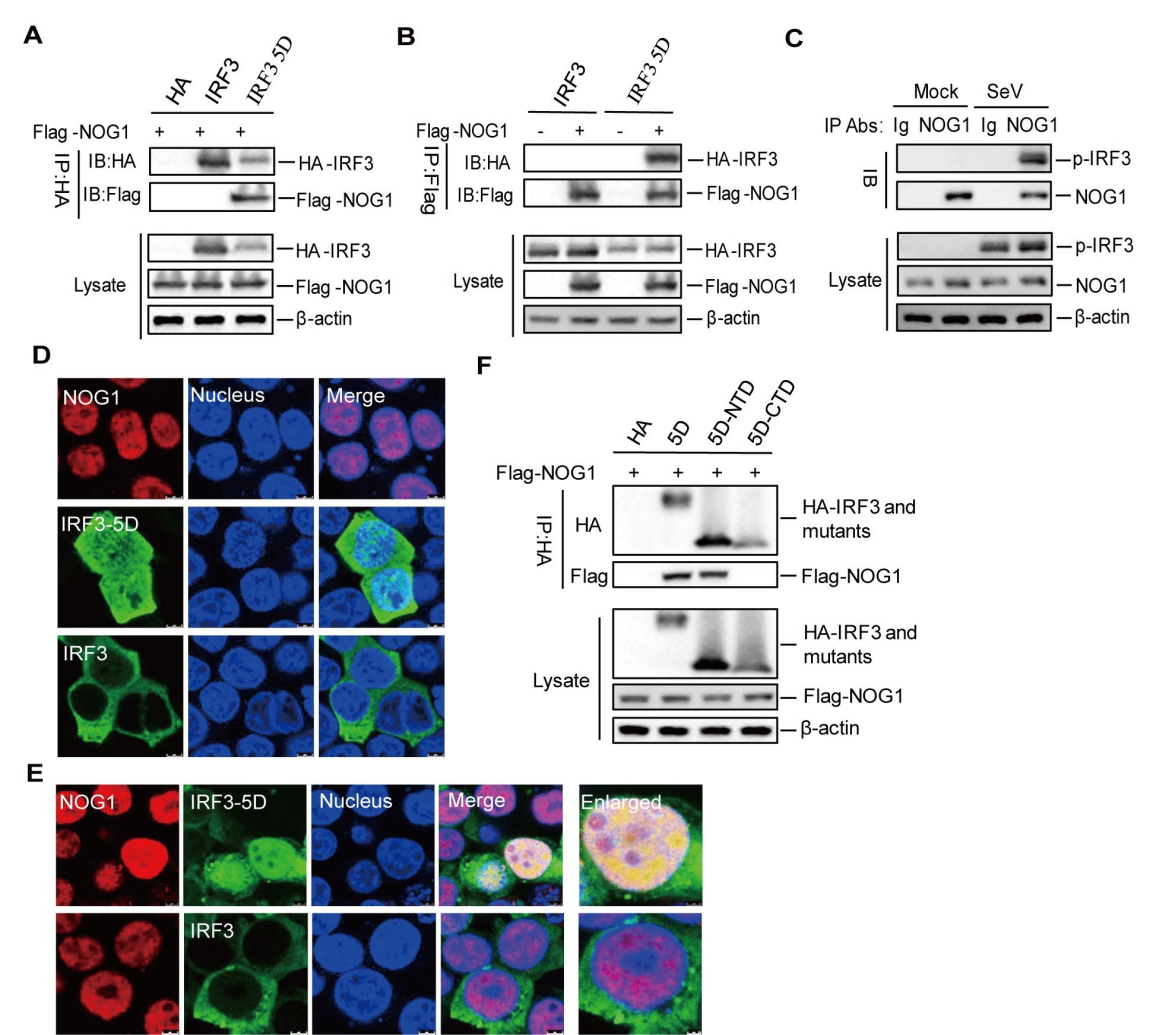
NOG1 interacts with phosphorylated IRF3. (A-B) HEK-293T cells were transfected with 3 μg of Flag-NOG1 expression plasmid along with 3 μg of HA empty vector, HA-IRF3, or HA-IRF3 5D expression plasmids. The cell lysates were immunoprecipitated with anti-HA antibody (A) or anti-Flag antibody (B) and subjected to Western blotting. (C) HEK-293T cells were mock-infected or infected with SeV for 12 h, and the cell lysates were immunoprecipitated with anti-NOG1 and anti-IgG antibodies and subjected to Western blotting. (D-E) HEK-293T cells were transfected with 0.5 μg of HA-IRF3 or HA-IRF3 5D expression plasmids for 24 h. The localization of endogenous NOG1, HA-IRF3, and HA-IRF3 5D was detected by IFA using anti-NOG1 and anti-HA antibodies. The Pearson’s co-localization coefficient was analyzed using ImageJ Software. (F) HEK-293T cells were transfected with 3 μg of Flag-NOG1 expression plasmid along with 3 μg of HA empty vector, HA-IRF3 5D, or HA-IRF3 5D mutants expression plasmids. The cell lysates were immunoprecipitated with anti-HA antibody and subjected to Western blotting.

To investigate how NOG1 suppresses IFN-β activation via IRF3, we explored a possible interaction between NOG1 and IRF3. HEK-293T cells were transfected with Flag-NOG1 expression plasmid along with HA empty vector, HA-IRF3, or HA-IRF3 5D expression plasmids. The cell lysates were immunoprecipitated with anti-HA antibody and subjected to Western blotting. HA-IRF3 5D but not HA-IRF3 pulled down Flag-NOG1 (Fig 6A). A reverse immunoprecipitation experiment was also performed using an anti-Flag antibody, which showed that Flag-NOG1 also immunoprecipitated HA-IRF3 5D (Fig 6B), suggesting that NOG1 interacted with activated IRF3. To confirm the endogenous interaction between NOG1 and the phosphorylated form of IRF3, HEK-293T cells were mock-infected or infected with SeV, and the cell lysates were immunoprecipitated with anti-NOG1 and anti-IgG antibodies and subjected to Western blotting. The endogenous NOG1 pulled down phosphorylated IRF3 (p-IRF3) (Fig 6C). Additionally, endogenous NOG1 also pulled down p-IRF3 in THP1 cells (S7 Fig).

The cellular localization of NOG1 and IRF3 was detected by indirect immunofluorescence assay (IFA), which showed that NOG1 and HA-IRF3 5D were mainly distributed in the nucleus, while HA-IRF3 was distributed in the cytoplasm (Fig 6D). The localization of NOG1 and IRF3 was further determined in cells transfected with NOG1 and HA-IRF3 or HA-IRF3 5D expression plasmids. Again, the endogenous NOG1 and HA-IRF3 5D were localized in the nucleus (Pearson’s co-localization coefficient: 0.87) (Fig 6E). The localization of NOG1 and IRF3 5D to the nucleus provides the permissible conditions for interaction.

We also identified the crucial region in IRF3 that was essential for NOG1-IRF3 interaction. The N-terminal domain (NTD, amino acids 1 to 197) and C-terminal domain (CTD, amino acids 198 to 427) of IRF3 were used to investigate the binding domain in IRF3 [29,30]. HEK-293T cells were transfected with Flag-NOG1 expression plasmid along with HA empty vector, HA-IRF3 5D, and HA-IRF3 5D mutants expression plasmids. The cell lysates were immunoprecipitated with anti-HA antibody. The results showed that IRF3 NTD, but not IRF3 CTD, interacted with NOG1 (Fig 6F). These results indicated that NOG1 interacted with p-IRF3 and that the IRF3 NTD was essential for the NOG1-IRF3 interaction.

### NOG1 impairs the DNA binding ability of IRF3

Upon viral infection, IRF3 is phosphorylated and forms dimers to enter the nucleus to activate IFN transcription. To determine the effect of NOG1 on IRF3 activation, the impact of NOG1 on IRF3 phosphorylation was investigated. WT and NOG1^+/-^ cells were mock-infected or infected with SeV, and the levels of IRF3 phosphorylation were detected by Western blotting. The results showed that neither the protein expression nor the phosphorylation levels of IRF3 were changed when NOG1 was decreased, indicating that NOG1 did not affect the IRF3 phosphorylation (S8A Fig). Subsequently, the impact of NOG1 on IRF3 nuclear translocation in SeV-infected cells was further detected by Western blotting. The IRF3 was predominantly in the cytoplasm in the mock-infected cells. Upon SeV stimulation, most IRF3 was transferred from the cytoplasm into the nucleus. However, there was no significant change in the level of IRF3 when NOG1 was overexpressed (S8B Fig). The abundance of IRF3 in SeV-infected cells was also quantified using ImageJ Software (S8B Fig, right). Furthermore, IFA results on these cells’ nuclear and cytoplasmic fractions were consistent with Western blotting results. In the mock-infected cells, the IRF3 was predominantly in the cytoplasm. In the SeV-infected cells, most control and NOG1-overexpressed cells displayed IRF3 nuclear localization, suggesting that IRF3 nuclear import is not affected by NOG1 (S8C Fig). These results demonstrated that NOG1 regulated IFN-β production without affecting the phosphorylation and nuclear translocation of IRF3.

**Fig 7:**
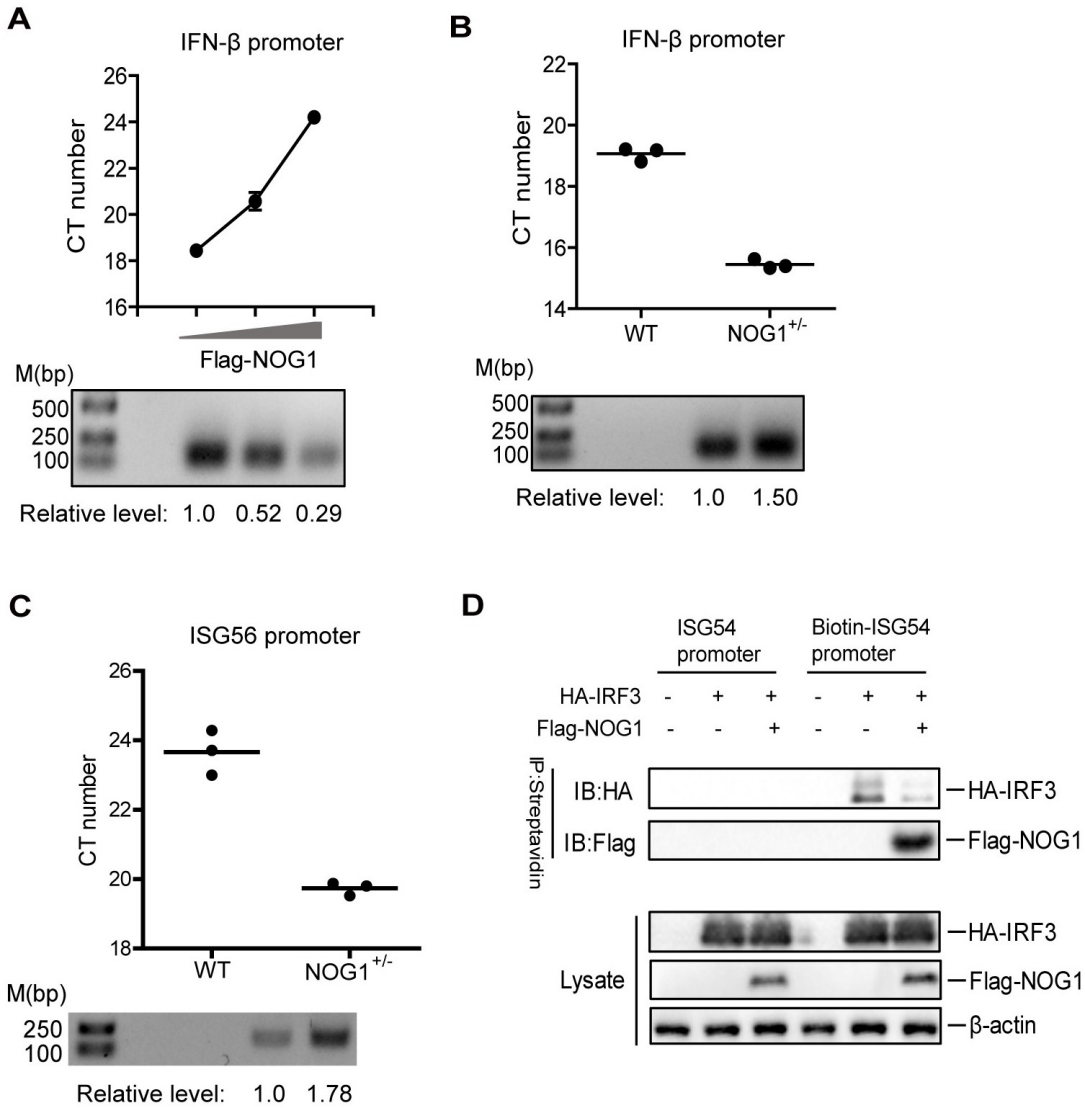
NOG1 impairs the DNA binding ability of IRF3. HEK-293T cells transfected with increasing Flag-NOG1 expression plasmid (0, 1, and 3 μg) (A), and WT and NOG1^+/-^ HEK-293T cells (B and C) were infected with SeV for 12 h. Chromatin was immunoprecipitated with an anti-IRF3 antibody. The impact of NOG1 on IRF3 binding onto IFN-β and ISG56 promoter was analyzed by quantitative ChIP assay. PCR products were analyzed by nucleic acid electrophoresis. The abundance of the immunoprecipitated DNA was normalized to the input DNA levels using ImageJ Software. (D) The effect of NOG1 on IRF3 binding onto ISG54 promoter. HEK-293T cells cotransfected with HA-IRF3 and Flag-NOG1 were infected with SeV for 12 h. Biotinylated and unbiotinylated promoters were mixed with the cell lysates. The streptavidin beads were then added and incubated, followed by washing with PBS. Western blotting was performed to detect IRF3 and NOG1.

Once IRF3 is in the nucleus, IRF3 binds to the promoter and ISRE sequence to activate the transcription of target genes [31]. In addition, NOG1 interacted with IRF3 NTD, which contains the DNA binding domain [15,32]. Therefore, we speculated that NOG1 might interfere with the DNA binding ability of IRF3. To confirm this speculation, the effect of NOG1 on IRF3 binding onto promoter was analyzed using chromatin immunoprecipitation (ChIP) assay and qPCR. PCR products were analyzed by nucleic acid electrophoresis, and the levels of immunoprecipitated DNA was normalized to the input DNA levels. No CT number was detected in the cells incubated with IgG. The CT number was increased, and the PCR production was decreased with the increase of NOG1 when the cells incubated with an anti-IRF3 antibody (Fig 7A and S9A Fig), suggesting that overexpression of NOG1 inhibited the interaction between IRF3 and the IFN-β promoter in a dose-dependent manner. Furthermore, the CT number was decreased, and the PCR production was increased in the NOG1^+/-^ cells compared to that in the WT cells, indicating that NOG1 deficiency promoted the binding of IRF3 and the IFN-β promoter (Fig 7B and S9B Fig). In addition, NOG1 deficiency also promoted the binding of IRF3 with IFN-β promoter in THP1 cells (S10A Fig).

ISG56 and ISG54 can be transcriptionally upregulated by activated IRF3 directly [33,34]. Thus, the impact of NOG1 on IRF3 binding onto ISG56 promoter was also analyzed using ChIP assay in HEK-293T cells. The results showed that the interaction between IRF3 and ISG56 promoter was enhanced in the NOG1^+/-^ cells compared to that in the WT cells (Fig 7C and S9C Fig), suggesting that NOG1 deficiency promoted the binding of IRF3 with ISG56 promoter.

ISG54 promoter can be recognized and bound by the activated IRF3 [15]. A DNA pulldown assay was performed by using biotinylated promoter oligonucleotide to detect the interaction between IRF3 and ISG54 promoter. As shown in Fig 7D, the IRF3 protein was pulled down by biotinylated promoter but not unbiotinyated promoter, and the amounts of IRF3 associated with the promoter were decreased in the presence of NOG1, which indicated that NOG1 prevents IRF3 binding onto the ISG54 promoter.

IRF3 binding to the target genes to activate their transcription requires interaction with CBP/p300 coactivators. Therefore, we further investigated the impact of NOG1 on the interaction between IRF3 and CBP in HEK-293T cells. The results showed that the endogenous CBP coprecipitated by IRF3 was not inhibited in the presence of NOG1, indicating that NOG1 does not affect the IRF3-CBP interaction (S10B Fig). Taken together, these results indicated that NOG1 attenuated the DNA binding capacity of IRF3, thereby suppressing the transcription of IFN-β and ISGs.

### The GTP binding domain of NOG1 is responsible for the inhibition of IFN-β

**Fig 8:**
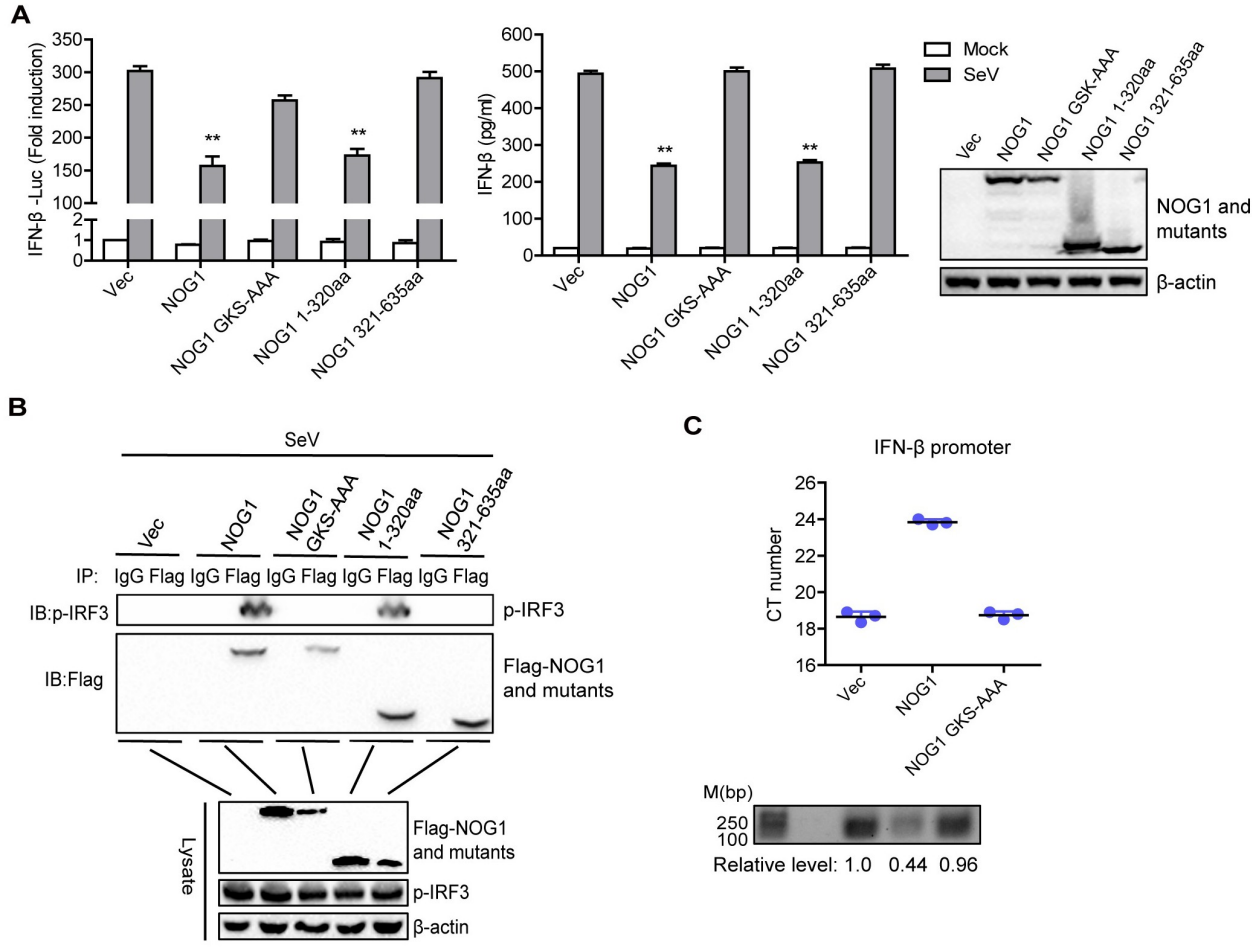
The GTP binding domain of NOG1 is responsible for the inhibition of IFN-β. (A) HEK-293T cells were transfected with 0.1 μg of IFN-β-Luc and 0.01 μg of pRL-TK plasmid along with 0.1 μg of Flag-NOG1- or Flag-NOG1-mutants-expressing plasmids (left). HEK-293T cells were transfected with 0.5 μg of Flag-NOG1- or Flag-NOG1-mutants-expressing plasmids (right). At 24 hpt, the cells were infected with SeV for 12 h. The promoter activation of IFN-β was determined by the dual-specific luciferase assay kit. The expression of IFN-β protein in the supernatant was detected by ELISA kit. The protein expression of NOG1 and its mutants was detected by Western blotting. (B) HEK-293T cells were transfected with 3 μg of Flag-NOG1- or Flag-NOG1-mutants-expressing plasmids. At 24 hpt, the cells were infected with SeV for 12 h. The cell lysates were immunoprecipitated with an anti-Flag antibody and subjected to Western blotting. (C) HEK-293T cells transfected with 3 μg of Flag-NOG1 or Flag-NOG1 GKS-AAA expression plasmid were infected with SeV for 12 h. The cells were collected and analyzed by quantitative ChIP assay. The abundance of PCR production was normalized to the input DNA levels using ImageJ Software.

The N-terminal region of NOG1 contains the GTP binding domain and is conserved from archaea to humans, while the C-terminal region of NOG1 is highly divergent in sequence in different organisms [17,35]. To assess which portion of NOG1 was required for the inhibition of innate immune responses, three mutants (residues 1–320 of NOG1, residues 321–635 of NOG1, and full-length protein mutated in the GTP binding domain of N-terminal region: GKS-AAA) were constructed, as described previously [17]. To explore the impact of NOG1 mutants on SeV-induced innate immune response, HEK-293T cells were transfected with IFN-β-Luc and pRL-TK plasmid along with Flag vector, Flag-NOG1, or Flag-NOG1 mutants expression plasmids. The promoter activity was detected by a Dual-Luciferase assay kit. Overexpression of NOG1 and NOG1 1-320aa significantly inhibited SeV-triggered activation of IFN-β promoter, while NOG1 GKS-AAA and NOG1 321-635aa did not affect the activity of IFN-β promoter (Fig 8A, left). Moreover, NOG1 GKS-AAA also lost its ability to inhibit SeV-induced IFN-β protein secretion (Fig 8A, right). The data indicated that the GTP binding domain in the N-terminal region of NOG1 was essential for the inhibition of IFN-β. The expression of NOG1 mutants was determined by Western blotting.

To identify the crucial region in NOG1 that was essential for NOG1-IRF3 interaction. HEK-293T cells were transfected with Flag-NOG1 or Flag-NOG1 mutants expression plasmid and then infected with SeV. The cell lysates were immunoprecipitated with an anti-Flag antibody and subjected to Western blotting. As a result, NOG1 and NOG1 1-320aa, but not NOG1 GKS-AAA and NOG1 321-635aa, pulled down p-IRF3 (Fig 8B), suggesting that NOG1 GKS-AAA abolished the ability to interact with IRF3. The results indicated that the GTP binding domain of NOG1 was essential for the NOG1-IRF3 interaction.

We further detected the impact of the GTP binding domain of NOG1 on the DNA binding ability of IRF3 by ChIP assay. As shown in Fig 8C and S9D Fig, overexpression of NOG1, but not NOG1 GKS-AAA, inhibited the interaction between IRF3 and the IFN-β promoter (PCR production was decreased in the NOG1-overexpressed cells). The relative level of PCR production was quantified using ImageJ Software. These results indicated that the GTP binding domain of NOG1 was essential for decreasing the DNA binding activity of IRF3 and inhibiting IFN-β.

## Discussion

RIG-like receptor (RLR)-, Toll-like receptor (TLR)-, NOD-like receptor (NLR)-, or cGAS-mediated innate immune response forms the first line of defense that protects hosts from invasion by RNA or DNA viruses. After virus infection, the IFN-β signal pathway is activated to induce IFN-β and ISG expression and initiate the appropriate adaptive immune response. The study of the mechanisms of innate immune response could contribute to better disease control or vaccine design. In the present study, we first investigated the roles of NOG1 in viral RNA- and DNA-mediated signaling pathways. Overexpression of NOG1 significantly inhibited SeV-, poly(I:C)-, or poly(dA:dT)-induced activation of IFN-β promoter, whereas the decrease in NOG1 had the opposite effects. Heterozygous knockout of NOG1 gene in mice indicates that NOG1 deficiency promotes RNA virus- and DNA virus-induced IFN-β protein and renders the mice more resistant to VSV and HSV-1 infection. This finding establishes a key role for NOG1 in the innate immune response.

Studies have identified that NOG1 involves various biological processes, including DNA mismatch repair system, cell cycle, 60 S ribosomal subunit biogenesis, glucose metabolism, and cancer [36–38]. Although NOG1 is required for 60 S ribosomal biogenesis, the cell viability and host proteins synthesis was not significantly affected in the NOG1^+/-^ cells compared to the WT cells. Additionally, the levels of IFN-β were increased and viral titers were decreased in the NOG1^+/-^ cells. These results suggested that NOG1-regulated IFN-β production was not associated with ribosomal biogenesis, revealing a novel biological function of NOG1. Upregulation of NOG1 has frequently been detected in many cancer cells [16,39]. Our data indicate that overexpression of NOG1 inhibits the innate immune response and promotes viral replication, which may be one of the reasons why many cancer cells are more susceptible to viral infection. In addition, the reduction of NOG1 induces cell cycle arrest in the G2/M period [16,19], which may be the reason why NOG1 knockout cells and mice are not viable.

IRF3, a key transcription factor, plays an important role in the induction of IFN-β and is essential for giving rise to the expression of many genes involved in the innate immune response [40]. After stimulation, cytoplasmic IRF3 is phosphorylated, forms dimers, and then enters the nucleus, where it interacts with the promoter of targeted genes, resulting in the transcription of IFN-I and the downstream ISGs [41]. Our results showed that NOG1 suppressed IRF3-5D-induced IFN-β promoter activity, suggesting that NOG1 may affect phosphorylation and the downstream events of IRF3. However, NOG1 did not affect the phosphorylation and nuclear import of IRF3, implying that NOG1 can regulate the function of IRF3 in the nucleus. As expected, NOG1 interacted with the phosphorylated IRF3 in the nucleus, and the DNA binding region of IRF3 was required for the NOG1-IRF3 interaction, which may impair IRF3 binding onto the promoter. The ChIP assay further verified that NOG1 inhibited the DNA binding ability of IRF3. Except for IRF3, IRF7 is also involved in the RLR-mediated signaling pathway [42,43], but NOG1 did not affect the function of IRF7, despite their structural and functional similarities. Thus, NOG1 specifically regulates the function of IRF3.

A variety of regulation mechanisms targeting IRF3 have been identified. For instance, foot-and-mouth disease virus (FMDV) and SVV infection inhibit type I IFN production by decreasing IRF3 protein expression [44,45]. HSV-1 ICP27 protein inhibits IRF3 phosphorylation and nuclear translocation by interacting with IRF3, leading to the inhibition of IFN-β production [46]. The Ebola virus suppresses the host’s innate immune response by blocking IRF3 dimerization and phosphorylation [47]. The nonessential accessory protein ML of the Thogoto virus inhibits host type I IFN production by blocking the interaction between IRF3 and CREB-binding protein [48]. Poxviruses N2 protein suppresses the activity of IRF3 in the nucleus [49], and the C6 protein prevents the transfer of IRF3 from the cytoplasm to the nucleus, inhibiting IFN-β production [50]. In addition, some intracellular proteins also regulate the function of IRF3. For instance, IRF1 interacts with IRF3 and enhances the activation of IRF3 by blocking the interaction between IRF3 and protein phosphatase 2A [51]; PRMT6 negatively regulates innate immunity by inhibiting IRF3 phosphorylation [52]; the deubiquitinase OTUD7B is a negative regulator of antiviral immunity by targeting IRF3 for selective autophagic degradation [53]; jumonji domain-containing protein 6 (JMJD6) negatively regulates RNA viruses-induced antiviral signaling by recruiting RNF5 to promote K48 ubiquitination of IRF3 [12]; and the cell growth-regulating nucleolar protein LYAR negatively regulates IFN-β-mediated immune responses by inhibiting the DNA binding ability of IRF3 [15]. Here, our results showed that the intracellular NOG1 protein interacted with activated IRF3 and disrupted the interaction of IRF3 and promotor, resulting in the inhibition of IFN-β production, which is similar to the mechanism by which LYAR inhibits IRF3 activation [15]. This finding reveals a novel function of NOG1 and broadens the regulation mechanisms targeting IRF3. Activation of IRF3 involves multiple processes including IRF3 protein expression, IRF3 phosphorylation and nuclear translocation, and the binding of IRF3 with promoters [54]. Although there are many negative regulators of IRF3, different proteins may negatively regulate IRF3 function at different processes. In addition, the proteins may express at different levels in different tissues, possibly leading to different proteins playing differentially principal antagonistic roles against IRF3 in different tissues.

The NTD of IRF3 contains the DNA binding domain [32], and the CTD of IRF3 includes the crucial region for its phosphorylation (containing the Ser385, Ser386, and Ser396 phosphorylation sites) [55]. NOG1 interacted with the NTD of IRF3, inhibiting the DNA binding activity of IRF3. Further detailed analysis revealed that the GTP binding domain of NOG1 was essential for the NOG1-IRF3 interaction, the decrease of DNA binding activity of IRF3, and the inhibition of IFN-β, revealing the importance of the GTP binding domain of NOG1 in innate immunity. Meanwhile, the results suggested that the NOG1-IRF3 interaction may be necessary for NOG1 to inhibit IFN-β production. In addition, previous results have shown that the GTP binding domain of NOG1 is required for stable association with the 60 s precursor [17,56]. Our results showed that the GTP binding domain of NOG1 is also essential for the inhibition of IFN-β, revealing the multiple functions of the GTP binding domain of NOG1.

**Fig 9:**
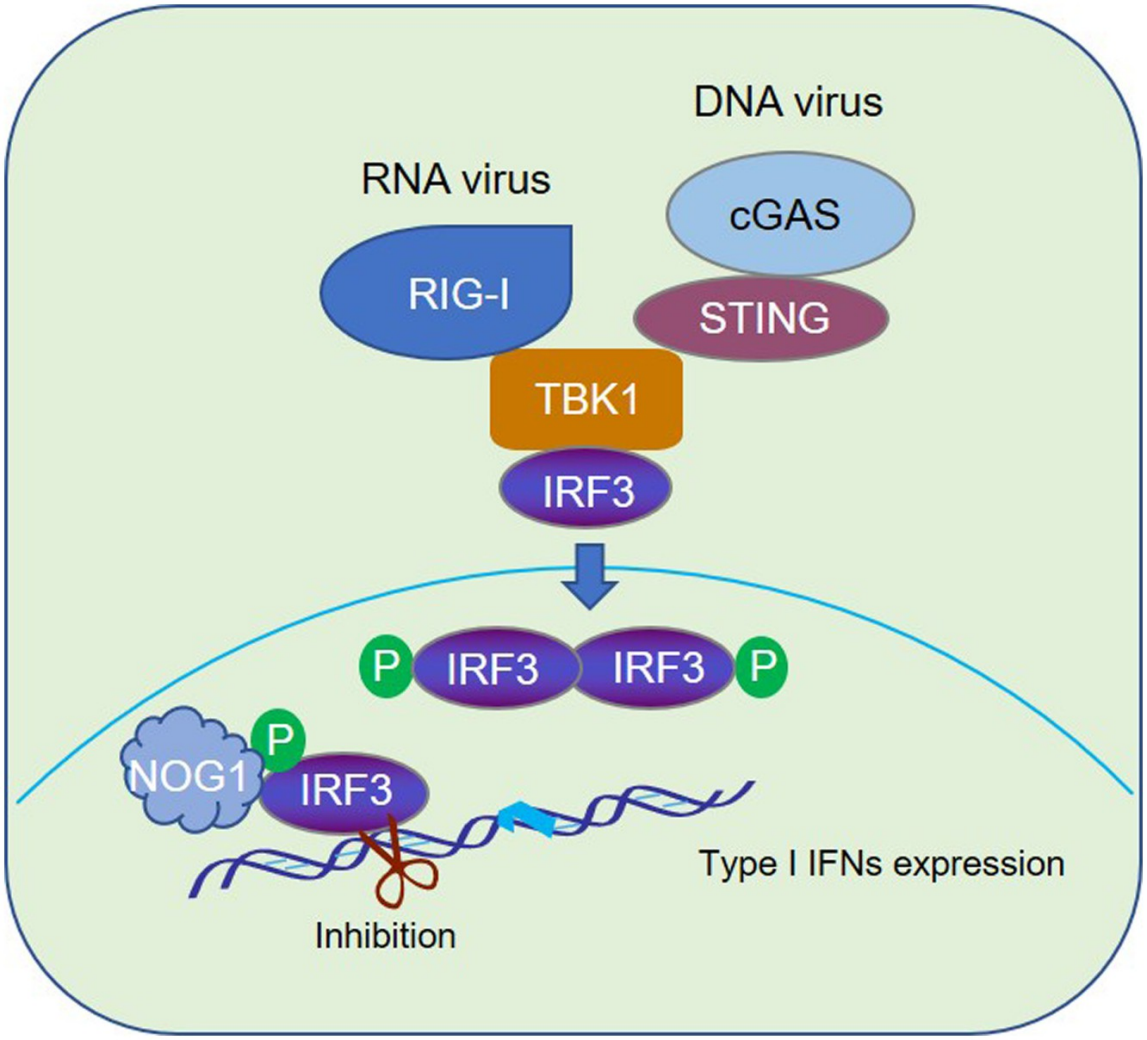
Schematic representation of the model of NOG1-mediated innate immune response. In this model, NOG1 negatively regulates RNA and DNA viruses-triggered innate immune response. NOG1 interacts with the phosphorylated IRF3 to impair its DNA binding activity, resulting in reduced production of IFN-β and its downstream ISGs, which in turn promotes viral replication. The GTP binding domain of NOG1 is responsible for this inhibitory effect.

Based on our findings, we proposed a model for the role of NOG1 in antiviral innate immune responses (Fig 9). NOG1 negatively regulates RNA and DNA viruses-triggered innate immune responses. NOG1 interacts with the phosphorylated IRF3 to impede its DNA binding activity, resulting in reduced production of IFN-β and its downstream ISGs, promoting viral replication. The GTP binding domain of NOG1 is responsible for this process. In conclusion, our study revealed an underlying mechanism of how NOG1 negatively regulates IFN-β by targeting IRF3, which would contribute to understanding the negative regulation of host innate immune responses and the function of NOG1 during virus infection.

## Materials and methods

### Ethics statement

All animals have handled strictly following good animal practice according to the Animal Ethics Procedures and Guidelines of the People’s Republic of China, and the study was approved by the Animal Ethics Committee of Lanzhou Veterinary Research Institute of the Chinese Academy of Agricultural Sciences (Licence no. SYXK (GAN) 2010–003).

### NOG1^+/-^ mice

NOG1^+/-^ mice (C57BL/6) were purchased from Cyagen Biosciences, China, and maintained in the specific pathogen-free (SPF) animal facility of Lanzhou Veterinary Research Institute with free access to food and water. Mouse experimental work was performed using 6~8-week-old mice.

NOG1^+/-^ mice were obtained using the CRISPR/Cas9 system. The single guide RNAs (sgRNAs) targeting exons 2, 3, and 4 were designed online (http://crispr.mit.edu/). The gRNAs and Cas9 were co-injected into fertilized mouse eggs to generate targeted knockout offspring. F0 founder animals were bred to WT mice to test germline transmission and F1 animal generation. The mouse NOG1 sgRNAs are: gRNA1: TGTAGGGGCTGAGCGATTGA-GGG; gRNA2: GATGATTACGAACGGATAAT-TGG; gRNA3: ATGCTCCCATTATCAGGGGC-AGG; gRNA4: TTTCAGGTGAGACAACTAAG-TGG. The exons 2, 3, and 4 of NOG1 were deleted.

### Detection of VSV and HSV-1 titers from mice

As described previously, VSV and HSV-1 from mice were isolated [57]. Briefly, to quantify the number of viral particles, part of the mouse tissue was weighed and homogenized by disposable tissue grinders (VWR, Radnor, PA) in Dulbecco’s modified Eagle medium (DMEM) (Thermo Scientific, Waltham, MA, USA) supplemented with 1% penicillin-streptomycin-neomycin antibiotic mixture, 2.5 μg/mL fungizone, and 1% L-Glutamax (Thermo Fisher Scientific). VSV and HSV-1 were titrated using Vero cells in DMEM media supplemented with 1% penicillin-streptomycin-neomycin antibiotic mixture, 2.5 μg/mL fungizone, and 1% L-Glutamax, as described previously [57].

### Histopathological and immunohistochemical analysis

The histopathological and immunohistochemical analyses were performed as described previously [58,59]. Briefly, the liver and spleen of mice were collected and fixed with 4% neutral formalin at room temperature for 5 d. Serial tissue sections were cut into 4-μm thicknesses after embedding in paraffin. The available slides were stained with hematoxylin and eosin (H & E). The histological changes were visualized by light microscopy (Olympus BX41, Olympus Optical Co., Tokyo, Japan).

The examination of VSV load in the mice’s liver was performed by immunohistochemical analysis. Paraffin-embedded microtome sections were incubated in xylene and serial dilutions of ethanol ranging from 100%-0% for 5 min at room temperature, with a final step of 3% H_2_O_2_ in methanol for 10 min. Then, the slides were incubated in sodium citrate buffer (PH6.0) maintained at 95°C for antigen retrieval. The available slides were incubated with an anti-GFP antibody (Servicebio, Wuhan, China) at 1:1000 dilution for 6 h at 4°C and further incubated with an avidin-conjugated secondary antibody (Servicebio) for 1 h, followed by incubation with biotinylated peroxidase (Servicebio) for an additional 1 h. Staining was visualized using the 3, 3-diaminobenzidine (DAB, Servicebio), according to the manufacturer′s protocol. The rate of positive cells was analyzed using the software AIpathwell (Servicebio).

### Cells and viruses

HEK-293 cells (ATCC CRL-1573), HEK-293T cells (ATCC CRL-11268), Vero cells (ATCC CCL-81), HeLa cells (ATCC CCL-2), IBRS-2 cells (ECACC 84100503), and A549 cells (ATCC CCL-185) were cultured in DMEM supplemented with 10% heat-inactivated fetal bovine serum (FBS, Gibco) and maintained at 37°C (5% CO_2_). THP1 cells (ATCC TIB-202) were cultured in RPMI 1640 medium containing 10% FBS. VSV-GFP strain was kindly provided by Prof. Bo Zhong (Wuhan University, China) and amplified in Vero cells. HSV-1 strain was kindly provided by Congcong Wang (Sun Yat-sen University, China) and amplified in Vero cells. SeV strain was kindly provided by Prof. Hongbing Shu (Wuhan University, China) and amplified in SPF eggs as described previously [60]. The SVV strain was isolated from cases of SVV in Guangdong Province, China, and stored in our laboratory [61], and virus propagation and titration were performed using IBRS-2 cells. SVV-GFP strain was prepared as described previously [62]. The 50% tissue culture infectious dose (TCID_50_) was calculated using Reed and Muench method.

### Plasmids and antibodies

The cDNA of human NOG1 was amplified from HEK-293T cells and cloned into p3xFlag-CMV-7.1 vector to yield the N terminal Flag-tagged expression construct (Flag-NOG1). Flag-NOG1 mutant expression plasmids were constructed by mutagenesis PCR. HA-RIG-I, HA-MDA5, HA-VISA, HA-TBK1, HA-IRF3, HA-IRF3 5D, and HA-IRF7 expression plasmids were stored by our laboratory previously. Carboxyl-terminal phosphorylation at amino acids 396, 398, 402, 404, and 405 cause the activation of IRF3. Thus, the phosphorylation mimicking mutant IRF3-5D is considered as a constitutively active form of IRF3 [63]. All constructed plasmids were analyzed and verified by DNA sequencing. The plasmids were transfected into cells using Lipofectamine 2000 (Thermo Scientific), according to the manufacturer′s protocol.

The commercial antibodies used in this study include anti-Flag polyclonal antibody (Sigma-Aldrich, St. Louis, MO), anti-Flag monoclonal antibody (Santa Cruz Biotechnology, Dallas, TX, USA), anti-Puromycin monoclonal antibody (Sigma-Aldrich), anti-IFN-β polyclonal antibody (Sigma-Aldrich), anti-NOG1 polyclonal antibody (Abcam, Cambridge, MA, USA), anti-HA polyclonal antibody (Abcam), anti-GFP polyclonal antibody (Cell Signaling Technology, Beverly, MA, USA), anti-IRF3 monoclonal antibody (Cell Signaling Technology), anti-p-IRF3 monoclonal antibody (Cell Signaling Technology), anti-RIG-I monoclonal antibody (Cell Signaling Technology), anti-TBK1 monoclonal antibody (Cell Signaling Technology), anti-MDA5 monoclonal antibody (Cell Signaling Technology), anti-MAVS monoclonal antibody (Cell Signaling Technology), anti-CBP monoclonal antibody (Cell Signaling Technology), anti-IL1β polyclonal antibody (Abclonal, Wuhan, China), and anti-β-actin monoclonal antibody (Sigma-Aldrich).

### Coimmunoprecipitation and Western blotting

HEK-293T cells were transfected with various indicated expressing plasmids. The cells were collected and lysed at the indicated time points using RIPA buffer containing protease inhibitors (Solarbio, Beijing, China). Then, the cells were immunoprecipitated with the indicated antibodies, as described previously [64]. The coimmunoprecipitation samples were resolved by SDS-PAGE for Western blotting and transferred to an Immobilon-P membrane (Millipore, Bedford, MA, USA). The membrane was blocked with 5% skim milk powder in TBST for 2 h at room temperature, then incubated with primary antibody (1:1000) for 6–8 h at 4°C and secondary antibody (1:10000) for 1 h at room temperature. The antibody-antigen complexes were visualized using chemiluminescence detection reagents (Thermo Fisher Scientific).

### Knockdown of NOG1 using siRNA

The small interfering RNAs (siRNAs) used in this study were designed and synthesized by Tsingke Biological Technology (Wuhan, China). Knockdown of endogenous NOG1 in THP1 and A549 cells was performed by transfection of NOG1 siRNA. NC siRNA was used as a negative control. According to the manufacturer’s protocol, the siRNA transfection was performed using Lipofectamine 2000. The human NOG1 sequences are F: GUGUUGACAUGGACGAUAA, R: UUAUCGUCCAUGUCAACAC.

### RNA extraction and quantitative PCR (qPCR)

Total RNAs in the cells were extracted by TRIzol reagent (Thermo Fisher Scientific). The extracted RNA was synthesized into cDNAs using HiScript II Q Select RT SuperMix (Vazyme, Nanjing, China). The expression of human IFN-β, ISG54, ISG56, and IL-1β mRNA was detected using the cDNA, ChamQ Universal SYBR qPCR Master Mix (Vazyme), and Mx3005P qPCR System (Agilent Technologies, Palo Alto, CA, USA). The *GAPDH* gene was used as an internal control. The relative expression of mRNA was calculated using the comparative cycle threshold (CT) (2^−ΔΔCT^) method [65]. The qPCR primers sequences are as follows:

human IFN-β-F: GACATCCCTGAGGAGATTAAG, R: ATGTTCTGGAGCATCTCATAG;
human ISG54-F: ACGGTATGCTTGGAACGATTG, R: AACCCAGAGTGTGGCTGATG;
human ISG56-F: CTTGAGCATCCTCGGGTTCATC, R: AAGTCAGCAGCCAGGTTTAGGG;
human IL-1β-F: CAAAGGCGGCCAGGATATAA, R: CTAGGGATTGAGTCCACATTCAG;
human GAPDH-F: CGGGAAGCTTGTGATCAATGG, R: GGCAGTGATGGCATGGACTG.

### Establishment of NOG1 knockout cell lines using CRISPR/Cas9 system

The NOG1 heterozygous knockout (NOG1^+/-^) cell lines were established using CRISPR/Cas9 system, as described previously [66]. The annealed single guide RNAs (sgRNAs) oligonucleotides targeting human NOG1 were designed by the online CRISPR design tool (http://crispr.mit.edu/) and cloned into lentiCRISPRv2. The human NOG1 sgRNA sequences are GTGGTGCCGTCCGCCAAGGT. Cells were transfected with lentiCRISPRv2-gRNA. The effectiveness of the designed sgRNA was evaluated using the T7 Endonuclease I. The cell lines were established by the limiting dilution in 96-well plates. Western blotting was performed to confirm NOG1 protein expression in the cell lines.

### Chromatin immunoprecipitation (ChIP)

The ChIP assay was performed using a pierce magnetic ChIP kit (Thermo Fisher Scientific) according to the manufacturer′s instructions. Briefly, cells were transfected with various plasmids, and WT and NOG1^+/-^ cells were infected with SeV. After cross-linking with 1% formaldehyde, the cells were harvested and resuspended using RIPA buffer. The chromatin was sheared into lengths of ∼300 bp by sonication. The lysates were incubated with anti-IRF3 antibody and protein G agarose. "IgG" immunoprecipitation was used as a negative control. The chromatin DNA was then eluted from the beads. Afterward, the bound DNA was extracted using phenol-chloroform and precipitated with ethanol after treatment with proteinase K. The quantity of DNA was determined by qPCR with specific primers. A comparative Ct method was used to assess the relative enrichment of the immunoprecipitated DNA. PCR products were analyzed by nucleic acid electrophoresis. The abundance of the immunoprecipitated DNA was normalized to the input DNA levels. The primers of the IFN-β promoter are as described previously [67]. The primer sequences of the ISG56 promoter are F: CAAATGCTGGCCAGTCATT, R: AAACAGCAGCCAATGGTGT.

### DNA pulldown assay

The DNA pulldown assay was performed as described previously [15]. The sequences of triple repeats of the ISG54 promoter are 5′-GGGAAAGTGAAA CTAGGGAAAGTGAAACTAGGGAAAGTGAAACTA-3′ [68,69]. DNA probes synthesized by Tsingke Biological Technology were labeled with biotin and used in the pulldown experiments. Briefly, HEK-293T cells transfected with the various plasmids were infected with SeV for 12 h. The cell lysates were mixed with DNA probes and incubated for 4 h at 4°C. The BeaverBeads streptavidin beads (Beyotime, Shanghai, China) were then added to the lysates for 2 h at 4°C. The beads were collected by magnetic separator and washed five times with PBS. Afterward, proteins that bound to the beads were resolved by SDS-PAGE.

### Luciferase reporter assay

HEK-293T cells were seeded in 24-well plates, and the monolayer cells were co-transfected with 0.1 μg/well of IFN-β-Luc, ISRE-Luc, or NF-ĸB Luc along with 0.01 μg/well of pRL-TK Renilla luciferase reporter plasmid and other plasmids. At 24 hpt, the cells were lysed, and the dual-specific luciferase assay kit (Promega Corporation, Wisconsin, USA) was used to analyze the firefly and Renilla luciferase activities, according to the manufacturer′s instruction.

### Indirect immunofluorescence assay

Indirect immunofluorescence assay (IFA) was performed as described in our previous study [70]. Briefly, cells cultured on Nunc glass bottom dishes (Thermo Fisher Scientific) were transfected with various plasmids or infected with SeV. The cells were fixed with an acetone/methanol mixture (1:1) for 24 h at 4°C and were blocked with 5% normal bovine serum for 6–8 h at 4°C. Then, cells were incubated with appropriate primary antibodies (1:250) overnight at 4°C and fluorochrome-conjugated secondary antibodies (1:500) in the dark for 6–8 h at 4°C. After that, the cells were stained with 4’,6-diamidino-2-phenylindole (DAPI) for 10 minutes at room temperature to show the nuclei. The fluorescence was visualized by a Nikon Eclipse 80i fluorescence microscope.

### Nuclear and cytoplasmic fractionation

HEK-293T cells (1×10^7^) were transfected with various plasmids and then infected with SeV. The cells were collected, and subcellular fractions were extracted using a Nuclear and cytoplasm extraction kit (Thermo Fisher Scientific) according to the manufacturer’s instructions.

### ELISA

The expression of IFN-β protein in the cells supernatant and mouse serum and tissue was detected by human or mouse IFN-β ELISA kit (Solarbio). The measured value was compared with the standard according to the manufacturer′s instructions.

### Puromycin labeling

The puromycin was used for monitoring host proteins synthesis, as described previously [70,71]. Briefly, WT and NOG1^+/-^ cells (10^6^ cells) were seeded in six-well plates. After 48 h incubation, the cells were labeled with 10 μg ml^-1^ of puromycin (Selleckchem, Houston, USA) for 30 min. The cells were then collected and analyzed by Western blotting using anti-puromycin and anti-β-actin antibodies.

### CCK-8 assay

CCK-8 assay was performed using CCK-8 Cytotoxicity Assay Kit (Solarbio) to detect cell viability. WT and NOG1^+/-^ cells (10^4^ cells) were seeded in 96-well plates with 100 μL medium for each well. After 48 h incubation, each well was incubated with 10 μL of CCK-8 solution for 2 h away from light before measuring the absorbance at 450 nm using a spectrofluorometer (Thermo Scientific).

### Statistical analysis

Statistical analysis was performed using SPSS Statistics for Windows, Version 17.0 (SPSS Inc., Chicago, IL, USA). The unpaired *t*-test (two-tailed test analysis) was used in this study. A *P-value <0.05 was considered statistically significant; A **P-value <0.01 was considered statistically significant. Data are presented as mean ± SD.

## Supporting information

S1 Fig The cell viability and host proteins synthesis in WT and NOG1^+/-^ cells. WT and NOG1^+/-^ cells were cultured in 96-well plates (A) or six-well plates (B) for 48 h. Then, the cells were incubated with 10 μL of CCK-8 solution for 2 h, followed by measuring the absorbance at 450 nm (A). The cells were labeled with 10 μg ml^-1^ of puromycin for 30 min and were analyzed by Western blotting using anti-puromycin antibody (B).
(TIF)

S2 Fig NOG1 did not affect the expression of IL-1β. (A) WT and NOG1^+/-^ HEK-293T cells were mock-infected or infected with SeV for 12 h. The mRNA expression of IL-1β was detected by qPCR. (B) THP1 cells transfected with increasing Flag-NOG1 expression plasmids were mock-infected or infected with SeV for 12 h, and mature IL-1β in supernatants (sup) or pro-IL-1β in lysates were determined by Western blotting.
(TIF)

S3 Fig The impact of NOG1 on innate immune responses in THP1 and A549 cells. THP1 and A549 cells transfected with 150 nM of NOG1 siRNA or NC siRNA were infected with HSV-1 (1 MOI) or VSV (1 MOI). The IFN-β protein in the supernatant was detected by ELISA kit. The expression of NOG1 protein in cells was detected by western blotting.
(TIF)

S4 Fig NOG1 promoted viral replication depending on IFN-β. (A) WT and NOG1^+/-^ HeLa cells were untreated or treated with an anti-IFN-β neutralizing antibody (1500, 2500, or 3000 units/mL) in DMEM with 1% FBS for 2 h. Then, the cells were infected with VSV (1 MOI) and incubated with the different dose of anti-IFN-β antibody for a further 16 h. Viral titers in the supernatant were measured by TCID_50_ assay. (B) IB (IBRS-2) cells transfected with increasing Flag-NOG1 expression plasmids were infected with SVV for 8 h, viral titers in the supernatant were measured by TCID_50_ assay.
(TIF)

S5 Fig The impact of NOG1 on VSV replication in mice. (A) WT and NOG1^+/-^ mice were intraperitoneally injected with VSV (6×10^7^ PFU) for 48 h. The liver of mice was collected and fixed with 4% neutral formalin. The VSV load in the liver was detected by immunohistochemical analysis using an anti-GFP antibody. The positive cells are indicated by a black arrowhead. The rate of positive cells was analyzed using the software AIpathwell. (B) WT and NOG1^+/-^ mice (n = 6) were intranasally injected with VSV (10^8^ PFU). The mortality of mice was recorded.
(TIF)

S6 Fig NOG1 did not interact with RIG-I, TBK1, MDA5, and MAVS. HEK-293T cells were transfected with 3 μg of Flag-NOG1 expression plasmid or empty vector. The cell lysates were immunoprecipitated with an anti-Flag antibody. The antibody-antigen complexes were visualized using anti-Flag, anti-RIG-I, anti-TBK1, anti-MDA5, and anti-MAVS antibodies.
(TIF)

S7 Fig NOG1 interacted with p-IRF3 in THP1 cells. THP1 cells were mock-infected or infected with SeV for 12 h, and the cell lysates were immunoprecipitated with anti-NOG1 and anti-IgG antibodies and subjected to Western blotting.
(TIF)

S8 Fig NOG1 did not affect the phosphorylation and nuclear translocation of IRF3. (A) WT and NOG1^+/-^ cells were mock-infected or infected with SeV for 12 h, the cells were collected, and the levels of IRF3 phosphorylation were detected by Western blotting. (B) HEK-293T cells were transfected with 3 μg of Flag-NOG1-expressing plasmid. At 24 hpt, the cells were mock-infected or infected with SeV for 12 h. Cells were harvested and subjected to nuclear and cytoplasmic fractionation. The expression of IRF3 in the cytoplasm (Cyt) and nucleus (Nuc) was detected by Western blotting, respectively. Histone 3.1 was used as a nuclear loading control and marker, and β-actin was used as a cytosolic loading control and marker. The abundance of IRF3 was quantified using ImageJ software. (C) HEK-293T cells transfected with 0.5 μg of Flag empty vector or Flag-NOG1-expressing plasmid were mock-infected or infected with SeV for 12 h. The subcellular localization of NOG1 and IRF3 was detected by IFA.
(TIF)

S9 Fig The input DNA levels for ChIP assay. A, B, C, and D represent the input DNA levels of Figs 7A, 7B, 7C and 8C, respectively.
(TIF)

S10 Fig NOG1 did not affect the interaction between IRF3 and CBP. (A) THP1 cells transfected with 150 nM of NOG1 or NC siRNA were immunoprecipitated with anti-IRF3 antibody. The impact of NOG1 on IRF3 binding onto IFN-β promoter was analyzed by quantitative ChIP assay. (B) HEK-293T cells transfected with Flag-NOG1 and/or HA-IRF3 expression plasmids were infected with SeV for 12 h, and the cell lysates were immunoprecipitated with anti-HA antibody and subjected to Western blotting.
(TIF)

## References

[1] Lian H, Zang R, Wei J, Ye W, Hu MM, Chen Y, et al. The Zinc-Finger Protein ZCCHC3 Binds RNA and Facilitates Viral RNA Sensing and Activation of the RIG-I-like Receptors. Immunity, 2018; 49: 438–448.e435. doi: 10.1016/j.immuni.2018.08.014 30193849

[2] Wang S, Sun X, Yi C, Zhang D, Lin X, Sun X, et al. AGO2 Negatively Regulates Type I Interferon Signaling Pathway by Competition Binding IRF3 with CBP/p300. Front Cell Infect Microbiol,2017; 7: 195. doi: 10.3389/fcimb.2017.00195 28589097 PMC5438986

[3] Chen S, Rong M. Regulation of cGAS activity by RNA-modulated phase separation. EMBO Rep, 2022; 24:e51800. doi: 10.15252/embr.202051800 36382803 PMC9900338

[4] Webb LG, Fernandez-Sesma A. RNA viruses and the cGAS-STING pathway: reframing our understanding of innate immune sensing. Curr Opin Virol, 2022; 53: 101206. doi: 10.1016/j.coviro.2022.101206 35180533

[5] Jurczyszak D, Manganaro L. ISG15 deficiency restricts HIV-1 infection. PLoS Pathog. 2022; 18: e1010405. doi: 10.1371/journal.ppat.1010405 35333911 PMC8986114

[6] Li J, Song J, Kang L, Huang L, Zhou S, Hu L, et al. pMGF505-7R determines pathogenicity of African swine fever virus infection by inhibiting IL-1β and type I IFN production. PLoS Pathog, 2021; 17: e1009733. 34310655 10.1371/journal.ppat.1009733 PMC8341718

[7] Ma C, Li Y, Zong Y, Velkov T, Wang C, Yang X, et al. p21 restricts influenza A virus by perturbing the viral polymerase complex and upregulating type I interferon signaling. PLoS Pathog. 2022; 18: e1010295. doi: 10.1371/journal.ppat.1010295 35180274 PMC8920271

[8] Xu J, Wang P, Li Z, Li Z, Han D, Wen M, et al. IRF3-binding lncRNA-ISIR strengthens interferon production in viral infection and autoinflammation. Cell Rep. 2021; 37: 109926. doi: 10.1016/j.celrep.2021.109926 34731629

[9] Liu H, Li C, He W, Chen J, Yang G, Chen L, et al. Free ISG15 inhibits Pseudorabies virus infection by positively regulating type I IFN signaling. PLoS Pathog. 2022; 18: e1010921. doi: 10.1371/journal.ppat.1010921 36315588 PMC9648840

[10] Chen K, Lai C, Su Y, Bao WD, Yang LN, Xu P, et al. cGAS-STING-mediated IFN-I Response in Host Defense and Neuroinflammatory Diseases. Curr Neuropharmacol. 2022; 20: 362–371. doi: 10.2174/1570159X19666210924110144 34561985 PMC9413793

[11] Belpaire A, van Geel N, Speeckaert R. From IL-17 to IFN-γ in inflammatory skin disorders: Is transdifferentiation a potential treatment target? Front Immunol, 2022; 13: 932265. 35967358 10.3389/fimmu.2022.932265 PMC9367984

[12] Zhang W, Wang Q, Yang F, Zhu Z, Duan Y, Yang Y, et al. JMJD6 negatively regulates cytosolic RNA induced antiviral signaling by recruiting RNF5 to promote activated IRF3 K48 ubiquitination. PLoS Pathog. 2021; 17: e1009366. doi: 10.1371/journal.ppat.1009366 33684176 PMC7971890

[13] Saitoh T, Tun-Kyi A, Ryo A, Yamamoto M, Finn G, Fujita T, et al. Negative regulation of interferon-regulatory factor 3-dependent innate antiviral response by the prolyl isomerase Pin1. Nat Immunol. 2006; 7: 598–605. doi: 10.1038/ni1347 16699525

[14] Kim JH, Kim TH, Lee HC, Nikapitiya C, Uddin MB. Rubicon Modulates Antiviral Type I Interferon (IFN) Signaling by Targeting IFN Regulatory Factor 3 Dimerization. J Virol. 2017; 91: e00248–17. doi: 10.1128/JVI.00248-17 28468885 PMC5487567

[15] Yang C, Liu X, Cheng T, Xiao R, Gao Q, Ming F, et al. LYAR Suppresses Beta Interferon Induction by Targeting Phosphorylated Interferon Regulatory Factor 3. J Virol. 2019; 93: e00769–19. doi: 10.1128/JVI.00769-19 31413131 PMC6803289

[16] Liu WB, Jia WD, Ma JL, Xu GL, Zhou HC, Peng Y, et al. Knockdown of GTPBP4 inhibits cell growth and survival in human hepatocellular carcinoma and its prognostic significance. Oncotarget. 2017; 8: 93984–93997. doi: 10.18632/oncotarget.21500 29212203 PMC5706849

[17] Jensen BC, Wang Q, Kifer CT, Parsons M. The NOG1 GTP-binding protein is required for biogenesis of the 60 S ribosomal subunit. J Biol Chem. 2003; 278: 32204–32211. doi: 10.1074/jbc.M304198200 12788953

[18] Zhou Q, Yin Y, Yu M, Gao D, Sun J, Yang Z, et al. GTPBP4 promotes hepatocellular carcinoma progression and metastasis via the PKM2 dependent glucose metabolism. Redox Biol. 2022; 56: 102458. doi: 10.1016/j.redox.2022.102458 36116159 PMC9483790

[19] Wu J, Chen G. GTPBP4: A New Therapeutic Target Gene Promotes Tumor Progression in Non-Small Cell Lung Cancer via EMT. J Oncol. 2022; 2022: 2164897. doi: 10.1155/2022/2164897 36405249 PMC9674418

[20] Hu Y, Xie J, Chen L, Tang Q, Wei W, Lin WF, et al. Integrated Analysis of Genomic and Transcriptomic Profiles Identified the Role of GTP Binding Protein-4 (GTPBP4) in Breast Cancer. Front Pharmacol. 2022; 13: 880445. doi: 10.3389/fphar.2022.880445 35784753 PMC9243593

[21] Chouchana L, Fernández-Ramos AA, Dumont F, Marchetti C, Ceballos-Picot I, Beaune P, et al. Molecular insight into thiopurine resistance: transcriptomic signature in lymphoblastoid cell lines. Genome Med. 2015; 7: 37. doi: 10.1186/s13073-015-0150-6 26015807 PMC4443628

[22] Reus JB, Trivino-Soto GS, Wu LI, Kokott K, Lim ES. SV40 Large T Antigen Is Not Responsible for the Loss of STING in 293T Cells but Can Inhibit cGAS-STING Interferon Induction. Viruses.2020; 12:137. doi: 10.3390/v12020137 31991682 PMC7077178

[23] Zang R, Lian H, Zhong X, Yang Q, Shu HB. ZCCHC3 modulates TLR3-mediated signaling by promoting recruitment of TRIF to TLR3. J Mol Cell Biol. 2020; 12: 251–262. doi: 10.1093/jmcb/mjaa004 32133501 PMC7232131

[24] Sui H, Zhou M, Imamichi H, Jiao X. STING is an essential mediator of the Ku70-mediated production of IFN-λ1 in response to exogenous DNA. Sci Signal. 2017; 10:eaah5054. 28720717 10.1126/scisignal.aah5054

[25] Lopez-Castejon G, Brough D. Understanding the mechanism of IL-1β secretion. Cytokine Growth Factor Rev. 2011; 22: 189–195. 22019906 10.1016/j.cytogfr.2011.10.001 PMC3714593

[26] Byun CS, Hwang S, Woo SH, Kim MY, Lee JS, Lee JI, et al. Adipose Tissue-Derived Mesenchymal Stem Cells Suppress Growth of Huh7 Hepatocellular Carcinoma Cells via Interferon (IFN)-β-Mediated JAK/STAT1 Pathway in vitro. Int J Med Sci. 2020; 17: 609–619. 32210710 10.7150/ijms.41354 PMC7085211

[27] Zhang X, Yang F, Li K, Cao W, Ru Y, Chen S, et al. The Insufficient Activation of RIG-I-Like Signaling Pathway Contributes to Highly Efficient Replication of Porcine Picornaviruses in IBRS-2 Cells. Mol Cell Proteomics. 2021; 20: 100147. doi: 10.1016/j.mcpro.2021.100147 34530158 PMC8503670

[28] Wang D, Fang L, Liu L, Zhong H, Chen Q, Luo R, et al. Foot-and-mouth disease virus (FMDV) leader proteinase negatively regulates the porcine interferon-λ1 pathway. Mol Immunol. 2011; 49: 407–412. 21975014 10.1016/j.molimm.2011.09.009

[29] Manivanh R, Mehrbach J, Knipe DM, Leib DA. Role of Herpes Simplex Virus 1 γ34.5 in the Regulation of IRF3 Signaling. J Virol. 2017; 91:e01156–17. 28904192 10.1128/JVI.01156-17 PMC5686731

[30] Liu H, Zhu Z, Feng T, Ma Z, Xue Q, Wu P, et al. African Swine Fever Virus E120R Protein Inhibits Interferon Beta Production by Interacting with IRF3 To Block Its Activation. J Virol. 2021; 95: e0082421. doi: 10.1128/JVI.00824-21 34190598 PMC8387055

[31] Weaver BK, Kumar KP, Reich NC. Interferon regulatory factor 3 and CREB-binding protein/p300 are subunits of double-stranded RNA-activated transcription factor DRAF1. Mol Cell Biol. 1998; 18: 1359–1368. doi: 10.1128/MCB.18.3.1359 9488451 PMC108849

[32] Honda K, Taniguchi T. IRFs: master regulators of signalling by Toll-like receptors and cytosolic pattern-recognition receptors. Nat Rev Immunol. 2006; 6: 644–658. doi: 10.1038/nri1900 16932750

[33] Kumthip K, Yang D. Pivotal role for the ESCRT-II complex subunit EAP30/SNF8 in IRF3-dependent innate antiviral defense. PLoS Pathog. 2017; 13: e1006713. doi: 10.1371/journal.ppat.1006713 29084253 PMC5679654

[34] Grandvaux N, Servant MJ, tenOever B, Sen GC, Balachandran S, Barber GN, et al. Transcriptional profiling of interferon regulatory factor 3 target genes: direct involvement in the regulation of interferon-stimulated genes. J Virol. 2002; 76: 5532–5539. doi: 10.1128/jvi.76.11.5532-5539.2002 11991981 PMC137057

[35] Klingauf-Nerurkar P, Gillet LC. The GTPase Nog1 co-ordinates the assembly, maturation and quality control of distant ribosomal functional centers. Elife. 2020; 9:e52474. doi: 10.7554/eLife.52474 31909713 PMC6968927

[36] Zhang Z, Wang J, Mao J, Li F, Chen W. Determining the Clinical Value and Critical Pathway of GTPBP4 in Lung Adenocarcinoma Using a Bioinformatics Strategy: A Study Based on Datasets from The Cancer Genome Atlas. Biomed Res Int. 2020; 2020: 5171242. doi: 10.1155/2020/5171242 33134380 PMC7593728

[37] Li L, Pang X, Zhu Z, Lu L, Yang J, Cao J. et al. GTPBP4 Promotes Gastric Cancer Progression via Regulating P53 Activity. Cell Physiol Biochem. 2018; 45: 667–676. doi: 10.1159/000487160 29408813

[38] Zhang N, Shen H, Huang S, Wang F, Liu H, Xie F, et al. LncRNA FGD5-AS1 functions as an oncogene to upregulate GTPBP4 expression by sponging miR-873-5p in hepatocellular carcinoma. Eur J Histochem. 2021; 65:3300. doi: 10.4081/ejh.2021.3300 34783233 PMC8611415

[39] Chen J, Zhang J, Zhang Z. Upregulation of GTPBP4 Promotes the Proliferation of Liver Cancer Cells. J Oncol. 2021; 2021: 1049104. doi: 10.1155/2021/1049104 34712323 PMC8548153

[40] Oduro PK, Zheng X, Wei J, Yang Y, Wang Y, Zhang H, et al. The cGAS-STING signaling in cardiovascular and metabolic diseases: Future novel target option for pharmacotherapy. Acta Pharm Sin B. 2022; 12: 50–75. doi: 10.1016/j.apsb.2021.05.011 35127372 PMC8799861

[41] Deater M, Tamhankar M. TDRD3 is an antiviral restriction factor that promotes IFN signaling with G3BP1. PLoS Pathog. 2022; 18: e1010249. doi: 10.1371/journal.ppat.1010249 35085371 PMC8824378

[42] Zhao M, Zhang Y, Yang X. Myeloid neddylation targets IRF7 and promotes host innate immunity against RNA viruses. PLoS Pathog. 2021; 17: e1009901. doi: 10.1371/journal.ppat.1009901 34506605 PMC8432861

[43] Chen S, Kumar S, Espada CE. N6-methyladenosine modification of HIV-1 RNA suppresses type-I interferon induction in differentiated monocytic cells and primary macrophages. PLoS Pathog. 2021; 17: e1009421. doi: 10.1371/journal.ppat.1009421 33690734 PMC7984636

[44] Xue Q, Liu H, Zhu Z, Yang F, Ma L, Cai X, et al. Seneca Valley Virus 3C(pro) abrogates the IRF3- and IRF7-mediated innate immune response by degrading IRF3 and IRF7. Virology. 2018; 518: 1–7. doi: 10.1016/j.virol.2018.01.028 29427864

[45] Wang D, Fang L, Luo R, Ye R, Fang Y, Xie L, et al. Foot-and-mouth disease virus leader proteinase inhibits dsRNA-induced type I interferon transcription by decreasing interferon regulatory factor 3/7 in protein levels. Biochem Biophys Res Commun. 2010; 399: 72–78. doi: 10.1016/j.bbrc.2010.07.044 20638368

[46] Guan X, Zhang M, Fu M, Luo S, Hu Q. Herpes Simplex Virus Type 2 Immediate Early Protein ICP27 Inhibits IFN-β Production in Mucosal Epithelial Cells by Antagonizing IRF3 Activation. Front Immunol. 2019; 10: 290. 30863402 10.3389/fimmu.2019.00290 PMC6399465

[47] Basler CF, Mikulasova A, Martinez-Sobrido L, Paragas J, Mühlberger E, Bray M, et al. The Ebola virus VP35 protein inhibits activation of interferon regulatory factor 3. J Virol. 2003; 77: 7945–7956. doi: 10.1128/jvi.77.14.7945-7956.2003 12829834 PMC161945

[48] Jennings S, Martínez-Sobrido L, García-Sastre A, Weber F, Kochs G. Thogoto virus ML protein suppresses IRF3 function. Virology. 2005; 331: 63–72. doi: 10.1016/j.virol.2004.10.015 15582653

[49] Ferguson BJ, Benfield CTO, Ren H, Lee VH, Frazer GL, Strnadova P, et al. Vaccinia virus protein N2 is a nuclear IRF3 inhibitor that promotes virulence. J Gen Virol. 2013; 94: 2070–2081. doi: 10.1099/vir.0.054114-0 23761407 PMC3749055

[50] Unterholzner L, Sumner RP, Baran M, Ren H, Mansur DS, Bourke NM, et al. Vaccinia virus protein C6 is a virulence factor that binds TBK-1 adaptor proteins and inhibits activation of IRF3 and IRF7. PLoS Pathog. 2011; 7: e1002247. doi: 10.1371/journal.ppat.1002247 21931555 PMC3169548

[51] Wang J, Li H, Xue B, Deng R, Huang X, Xu Y, et al. IRF1 Promotes the Innate Immune Response to Viral Infection by Enhancing the Activation of IRF3. J Virol. 2020; 94:e01231–20. doi: 10.1128/JVI.01231-20 32878885 PMC7592201

[52] Jiang Z, Cheng X, Sun Z, Hu J, Xu X, Li M, et al. Grass carp PRMT6 negatively regulates innate immunity by inhibiting the TBK1/IRF3 binding and cutting down IRF3 phosphorylation level. Dev Comp Immunol. 2022; 129: 104351. doi: 10.1016/j.dci.2022.104351 35033573

[53] Xie W, Tian S, Yang J, Cai S, Jin S, Zhou T, et al. OTUD7B deubiquitinates SQSTM1/p62 and promotes IRF3 degradation to regulate antiviral immunity. Autophagy. 2022; 18: 2288–2302. doi: 10.1080/15548627.2022.2026098 35100065 PMC9542415

[54] Al Hamrashdi M, Brady G. Regulation of IRF3 activation in human antiviral signaling pathways. Biochem Pharmacol. 2022; 200: 115026. doi: 10.1016/j.bcp.2022.115026 35367198

[55] Jing T, Zhao B, Xu P. The Structural Basis of IRF-3 Activation upon Phosphorylation. J Immunol. 2020; 205: 1886–1896. doi: 10.4049/jimmunol.2000026 32826280 PMC7511445

[56] Honma Y, Kitamura A, Shioda R, Maruyama H, Ozaki K, Oda Y, et al. TOR regulates late steps of ribosome maturation in the nucleoplasm via Nog1 in response to nutrients. EMBO J. 2006; 25: 3832–3842. doi: 10.1038/sj.emboj.7601262 16888624 PMC1553199

[57] Chen H, Humes ST, Rose M, Robinson SE, Loeb JC, Sabaraya IV, et al. Hydroxyl functionalized multi-walled carbon nanotubes modulate immune responses without increasing 2009 pandemic influenza A/H1N1 virus titers in infected mice. Toxicol Appl Pharmacol. 2020; 404: 115167. doi: 10.1016/j.taap.2020.115167 32771490 PMC10636740

[58] Zhang S, Hu B, Xu J, Ren Q, Wang L, Wang S, et al. Influenza A virus infection induces liver injury in mice. Microb Pathog. 2019; 137: 103736. doi: 10.1016/j.micpath.2019.103736 31505263 PMC7125922

[59] Fensterl V, Wetzel JL, Ramachandran S, Ogino T, Stohlman SA, Bergmann CC, et al. Interferon-induced Ifit2/ISG54 protects mice from lethal VSV neuropathogenesis. PLoS Pathog. 2012; 8: e1002712. doi: 10.1371/journal.ppat.1002712 22615570 PMC3355090

[60] Zhang W, Yang F, Zhu Z, Yang Y, Wang Z, Cao W, et al. Cellular DNAJA3, a Novel VP1-Interacting Protein, Inhibits Foot-and-Mouth Disease Virus Replication by Inducing Lysosomal Degradation of VP1 and Attenuating Its Antagonistic Role in the Beta Interferon Signaling Pathway. J Virol. 2019; 93:e00588–19. doi: 10.1128/JVI.00588-19 30996089 PMC6580959

[61] Zhu Z, Yang F, Chen P, Liu H, Cao W, Zhang K, et al. Emergence of novel Seneca Valley virus strains in China, 2017. Transbound Emerg Dis. 2017; 64: 1024–1029. doi: 10.1111/tbed.12662 28544501

[62] Poirier JT, Reddy PS, Idamakanti N, Li SS, Stump KL, Burroughs KD, et al. Characterization of a full-length infectious cDNA clone and a GFP reporter derivative of the oncolytic picornavirus SVV-001. J Gen Virol. 2012; 93: 2606–2613. doi: 10.1099/vir.0.046011-0 22971818

[63] Wang JT, Chang LS, Chen CJ, Doong SL, Chang CW, Chen MR, et al. Glycogen synthase kinase 3 negatively regulates IFN regulatory factor 3 transactivation through phosphorylation at its linker region. Innate Immun. 2014; 20: 78–87. doi: 10.1177/1753425913485307 23685991

[64] Li D, Yang W, Yang F, Liu H, Zhu Z, Lian K, et al. The VP3 structural protein of foot-and-mouth disease virus inhibits the IFN-beta signaling pathway. FASEB J. 2016; 30: 1757–1766. 26813975 10.1096/fj.15-281410

[65] Schmittgen TD, Livak KJ. Analyzing real-time PCR data by the comparative C(T) method. Nat Protoc. 2008; 3: 1101–1108. doi: 10.1038/nprot.2008.73 18546601

[66] Jin J, Xu Y, Huo L, Ma L, Scott AW, Pizzi MP, et al. An improved strategy for CRISPR/Cas9 gene knockout and subsequent wildtype and mutant gene rescue. PLoS One. 2020; 15: e0228910. doi: 10.1371/journal.pone.0228910 32053639 PMC7018052

[67] Li X, Guo G, Lu M, Chai W, Li Y, Tong X, et al. Long Noncoding RNA Lnc-MxA Inhibits Beta Interferon Transcription by Forming RNA-DNA Triplexes at Its Promoter. J Virol. 2019; 93:e00786–19. doi: 10.1128/JVI.00786-19 31434735 PMC6803265

[68] Nakaya T, Sato M, Hata N, Asagiri M, Suemori H, Noguchi S, et al. Gene induction pathways mediated by distinct IRFs during viral infection. Biochem Biophys Res Commun. 2001; 283: 1150–1156. doi: 10.1006/bbrc.2001.4913 11355893

[69] Meng F, Zhou R, Wu S, Zhang Q, Jin Q, Zhou Y, et al. Mst1 shuts off cytosolic antiviral defense through IRF3 phosphorylation. Genes Dev. 2016; 30: 1086–1100. doi: 10.1101/gad.277533.116 27125670 PMC4863739

[70] Liu H, Xue Q, Cao W, Yang F, Ma L, Liu WJ, et al. Foot-and-mouth disease virus nonstructural protein 2B interacts with cyclophilin A, modulating virus replication. FASEB J. 2018; fj201701351. doi: 10.1096/fj.201701351 29906248

[71] Schmidt EK, Clavarino G, Ceppi M, Pierre P. SUnSET, a nonradioactive method to monitor protein synthesis. Nat Methods. 2009. 6: 275–277. doi: 10.1038/nmeth.1314 19305406

