# Supplementary figures and images for: NOG1 downregulates type I interferon production by targeting phosphorylated interferon regulatory factor 3
Source: PLoS Pathog. 2023 Jul 6;19(7):e1011511. doi: 10.1371/journal.ppat.1011511 (PMC10353805; doi:10.1371/journal.ppat.1011511)

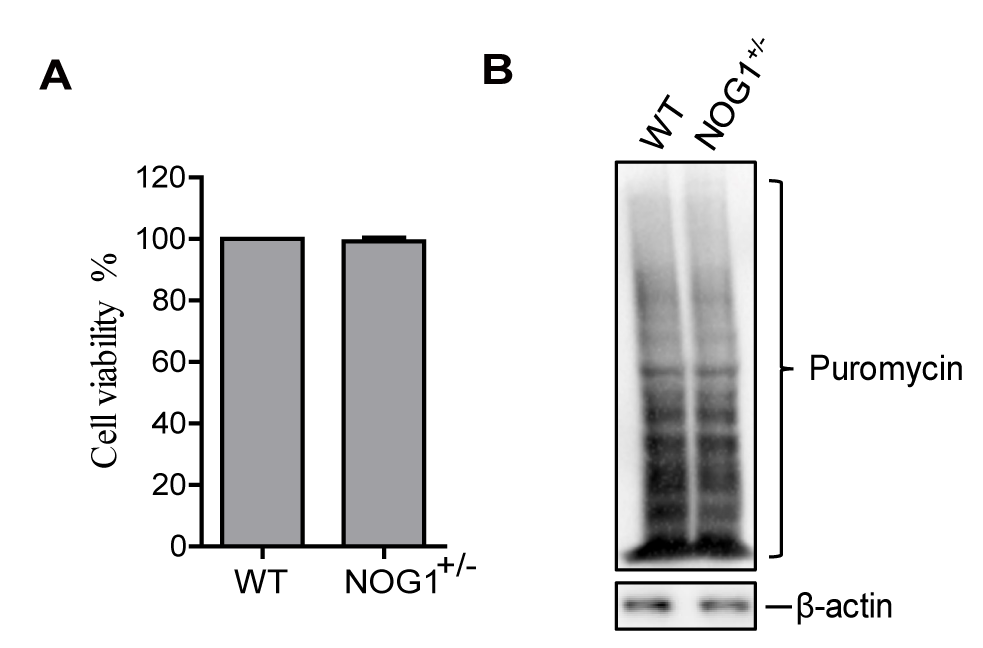

Supplement: S1 Fig — WT and NOG1+/- cells were cultured in 96-well plates (A) or six-well plates (B) for 48 h. Then, the cells were incubated with 10 μL of CCK-8 solution for 2 h, followed by measuring the absorbance at 450 nm (A). The cells were labeled with 10 μg ml-1 of puromycin for 30 min and were analyzed by Western blotting using anti-puromycin antibody (B). (TIF) [file ppat.1011511.s001.tif]

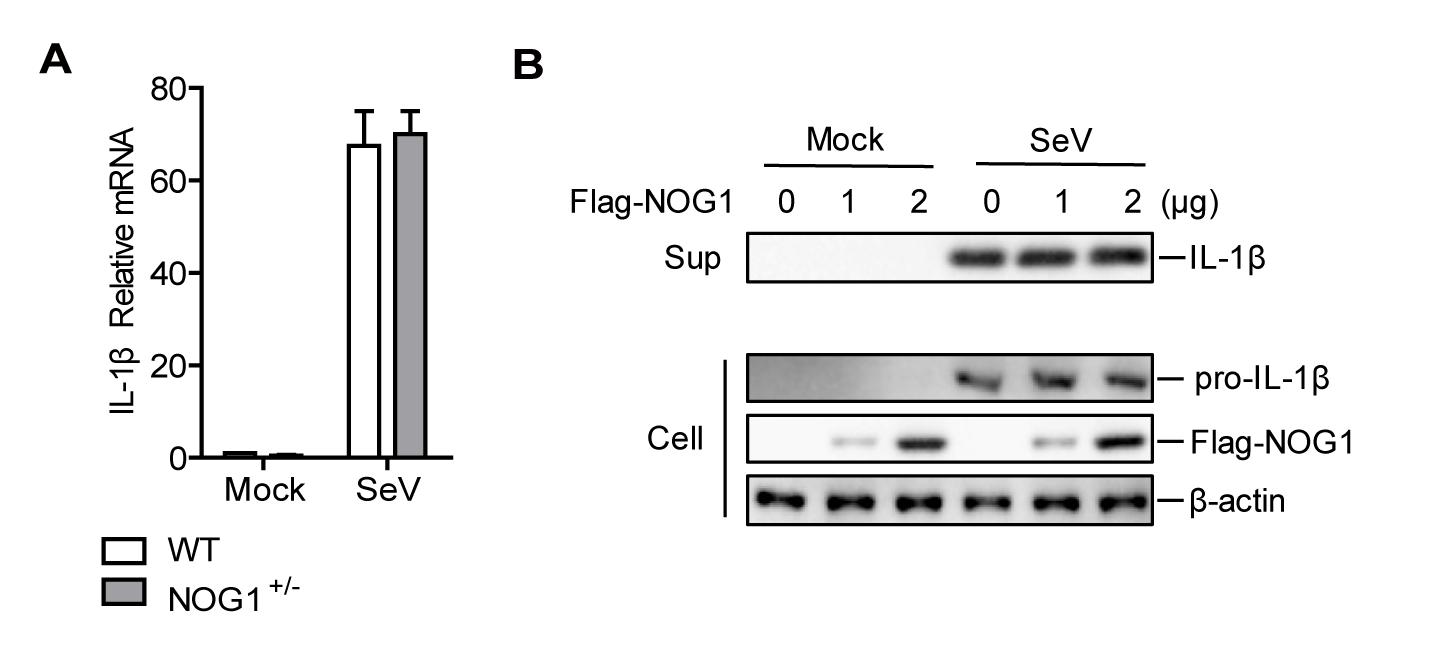

Supplement: S2 Fig — (A) WT and NOG1+/- HEK-293T cells were mock-infected or infected with SeV for 12 h. The mRNA expression of IL-1β was detected by qPCR. (B) THP1 cells transfected with increasing Flag-NOG1 expression plasmids were mock-infected or infected with SeV for 12 h, and mature IL-1β in supernatants (sup) or pro-IL-1β in lysates were determined by Western blotting. (TIF) [file ppat.1011511.s002.tif]

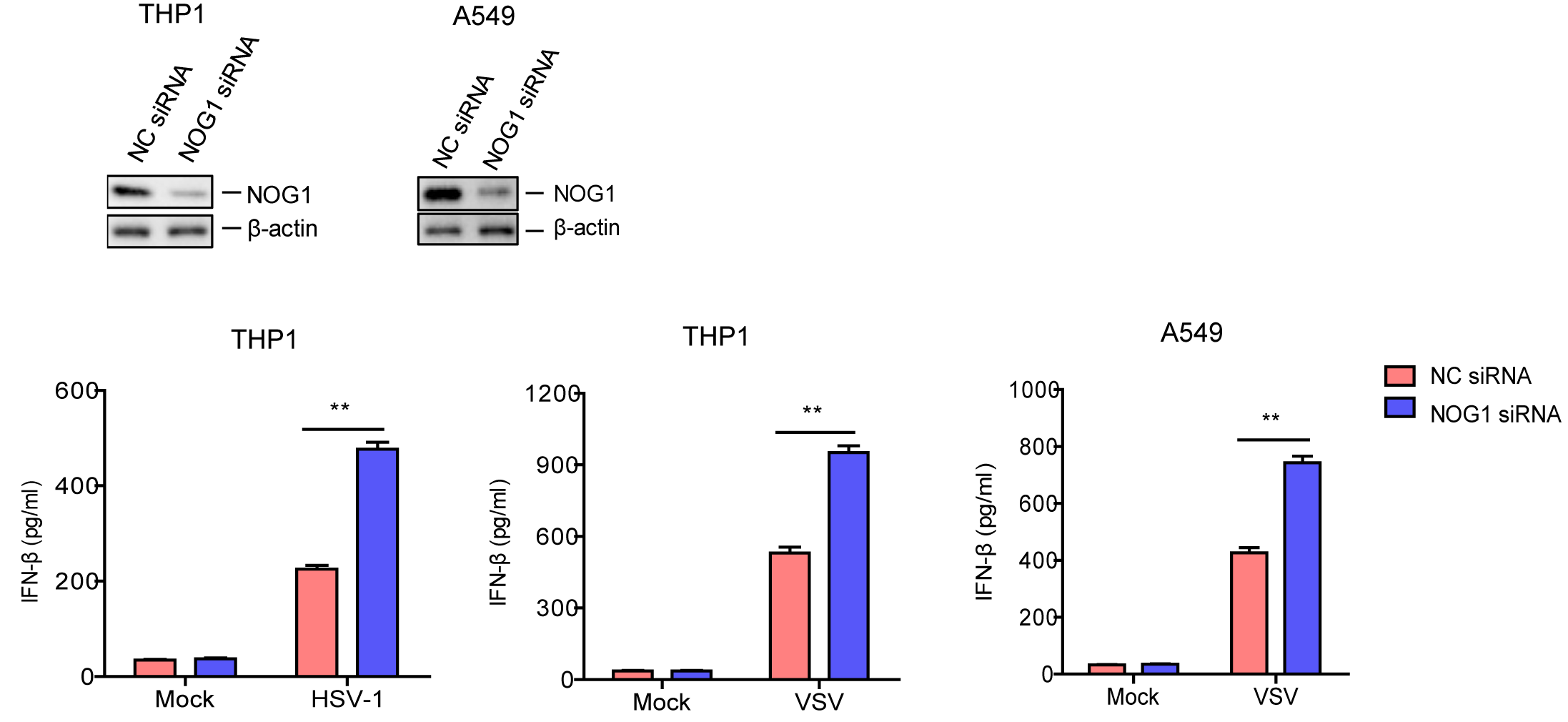

Supplement: S3 Fig — THP1 and A549 cells transfected with 150 nM of NOG1 siRNA or NC siRNA were infected with HSV-1 (1 MOI) or VSV (1 MOI). The IFN-β protein in the supernatant was detected by ELISA kit. The expression of NOG1 protein in cells was detected by western blotting. (TIF) [file ppat.1011511.s003.tif]

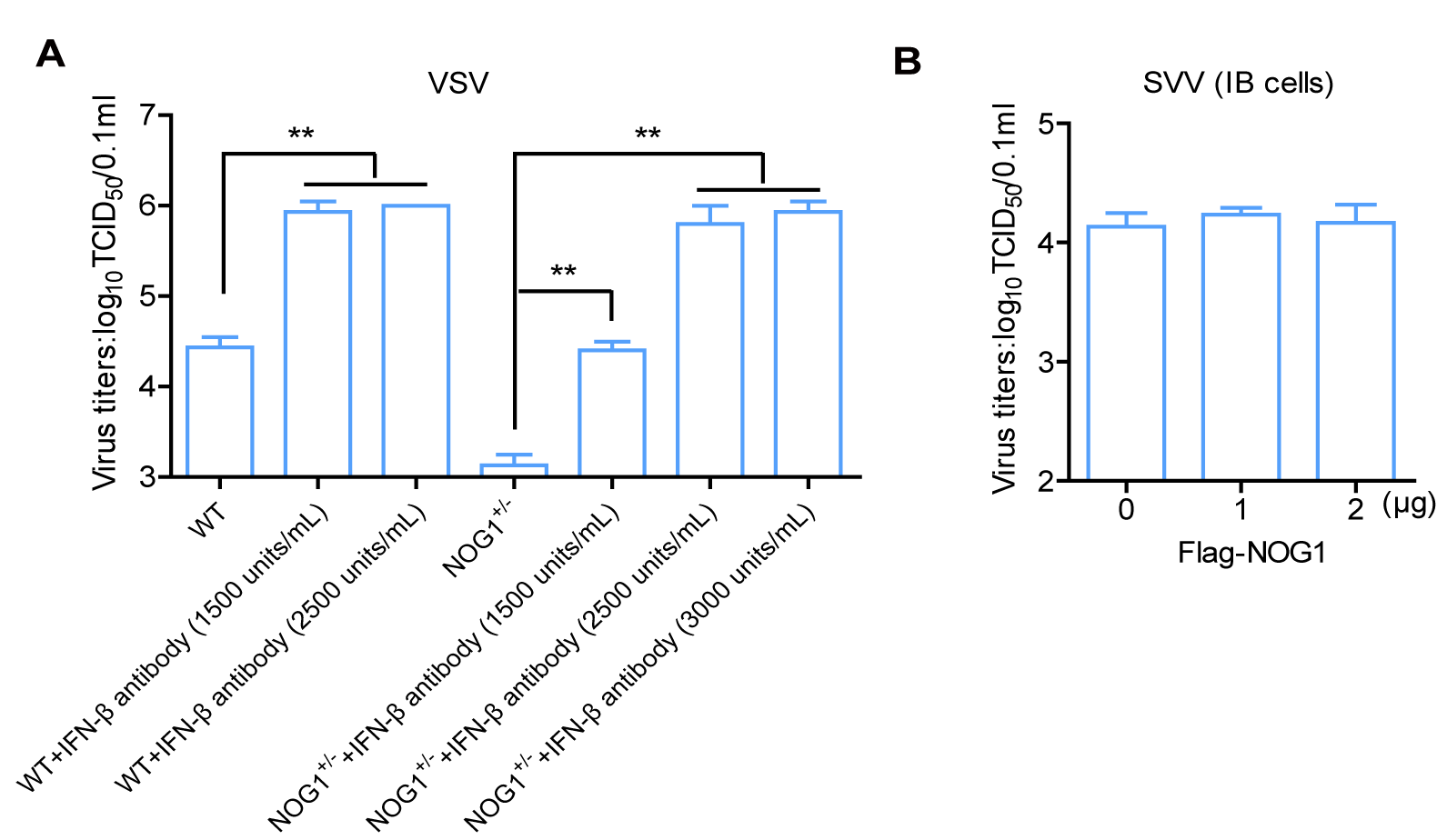

Supplement: S4 Fig — (A) WT and NOG1+/- HeLa cells were untreated or treated with an anti-IFN-β neutralizing antibody (1500, 2500, or 3000 units/mL) in DMEM with 1% FBS for 2 h. Then, the cells were infected with VSV (1 MOI) and incubated with the different dose of anti-IFN-β antibody for a further 16 h. Viral titers in the supernatant were measured by TCID50 assay. (B) IB (IBRS-2) cells transfected with increasing Flag-NOG1 expression plasmids were infected with SVV for 8 h, viral titers in the supernatant were measured by TCID50 assay. (TIF) [file ppat.1011511.s004.tif]

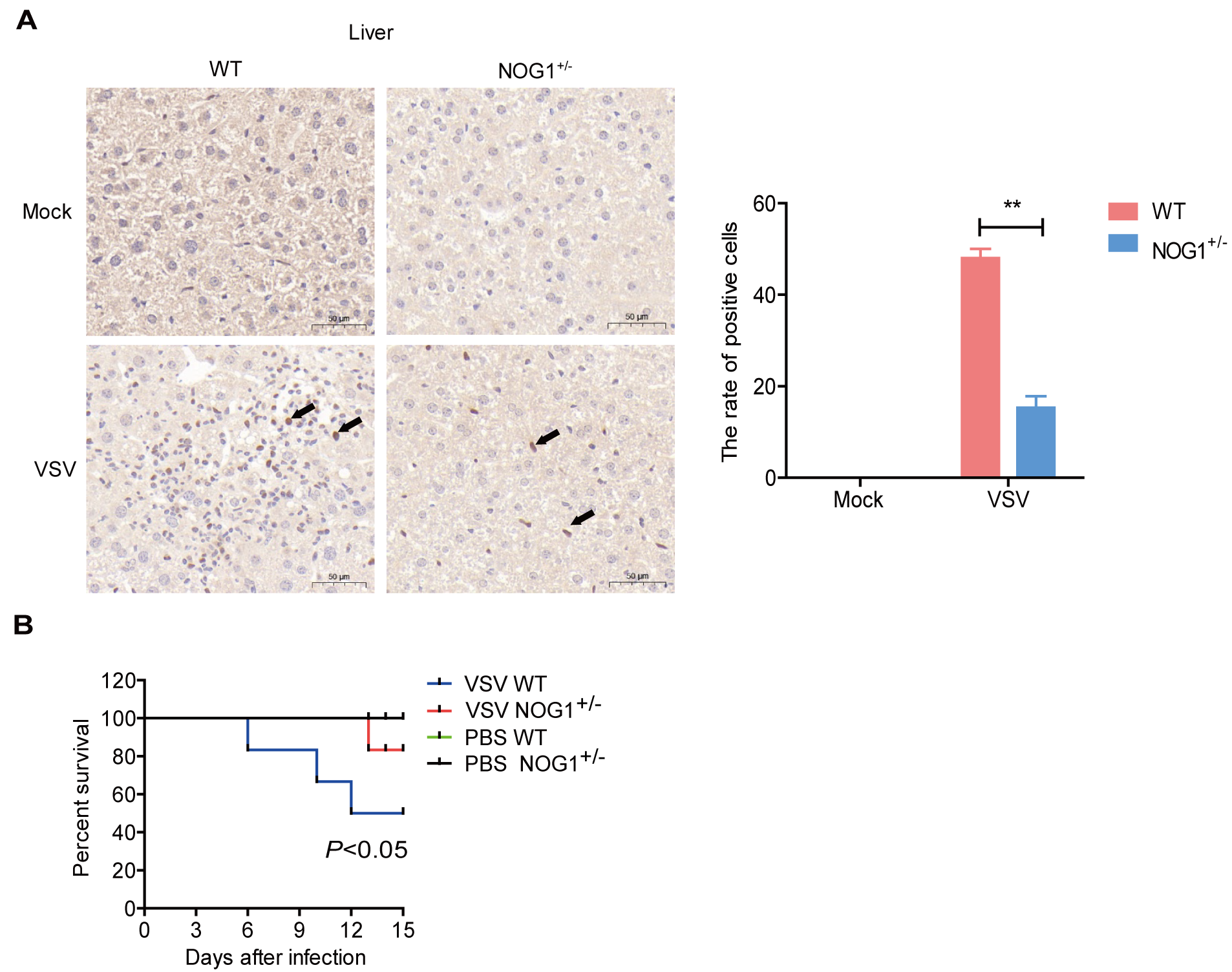

Supplement: S5 Fig — (A) WT and NOG1+/- mice were intraperitoneally injected with VSV (6×10^7^ PFU) for 48 h. The liver of mice was collected and fixed with 4% neutral formalin. The VSV load in the liver was detected by immunohistochemical analysis using an anti-GFP antibody. The positive cells are indicated by a black arrowhead. The rate of positive cells was analyzed using the software AIpathwell. (B) WT and NOG1+/- mice (n = 6) were intranasally injected with VSV (108 PFU). The mortality of mice was recorded. (TIF) [file ppat.1011511.s005.tif]

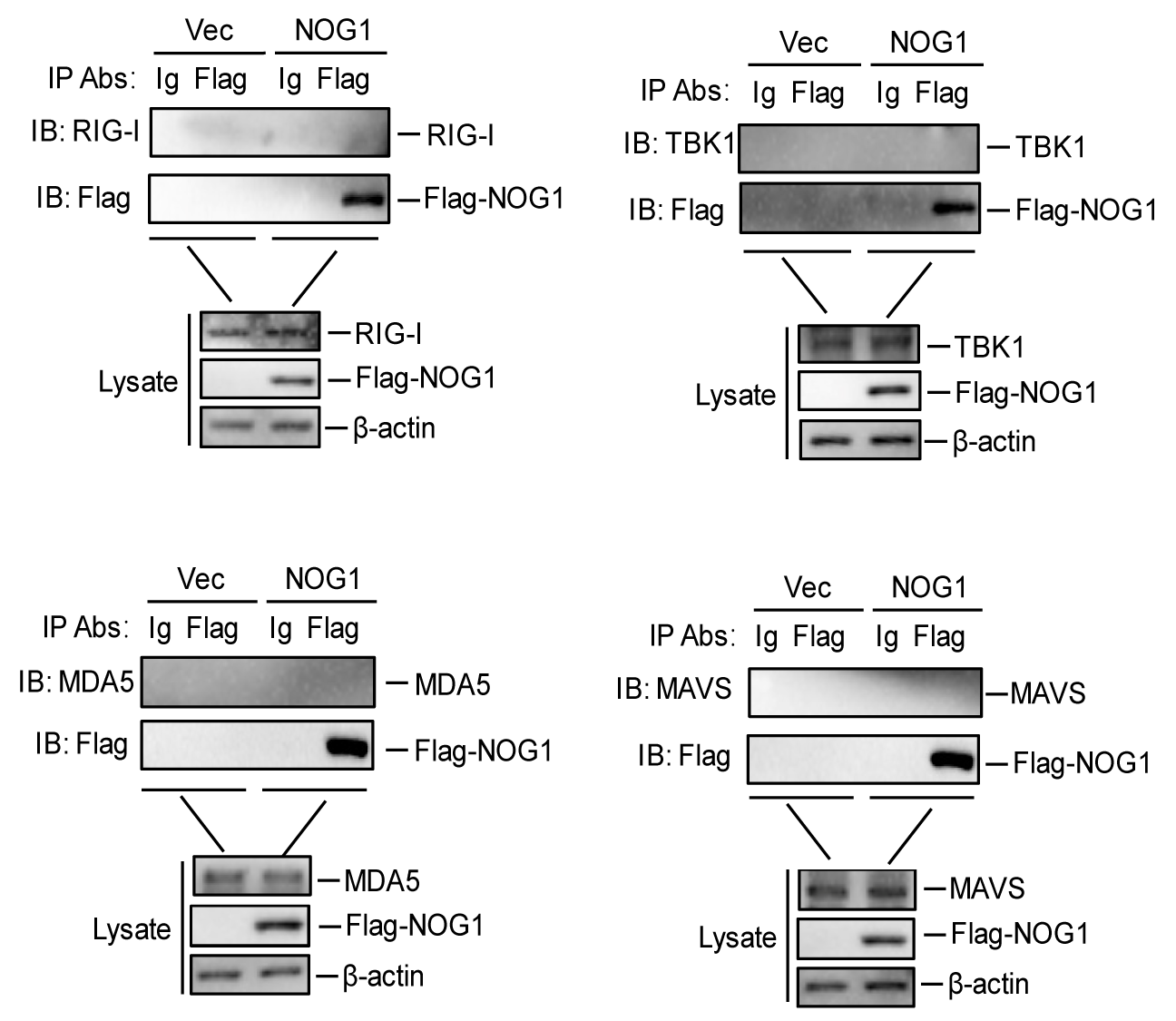

Supplement: S6 Fig — HEK-293T cells were transfected with 3 μg of Flag-NOG1 expression plasmid or empty vector. The cell lysates were immunoprecipitated with an anti-Flag antibody. The antibody-antigen complexes were visualized using anti-Flag, anti-RIG-I, anti-TBK1, anti-MDA5, and anti-MAVS antibodies. (TIF) [file ppat.1011511.s006.tif]

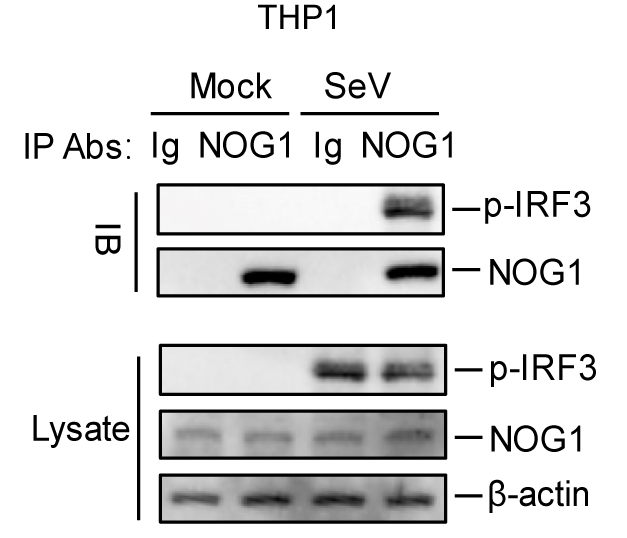

Supplement: S7 Fig — THP1 cells were mock-infected or infected with SeV for 12 h, and the cell lysates were immunoprecipitated with anti-NOG1 and anti-IgG antibodies and subjected to Western blotting. (TIF) [file ppat.1011511.s007.tif]

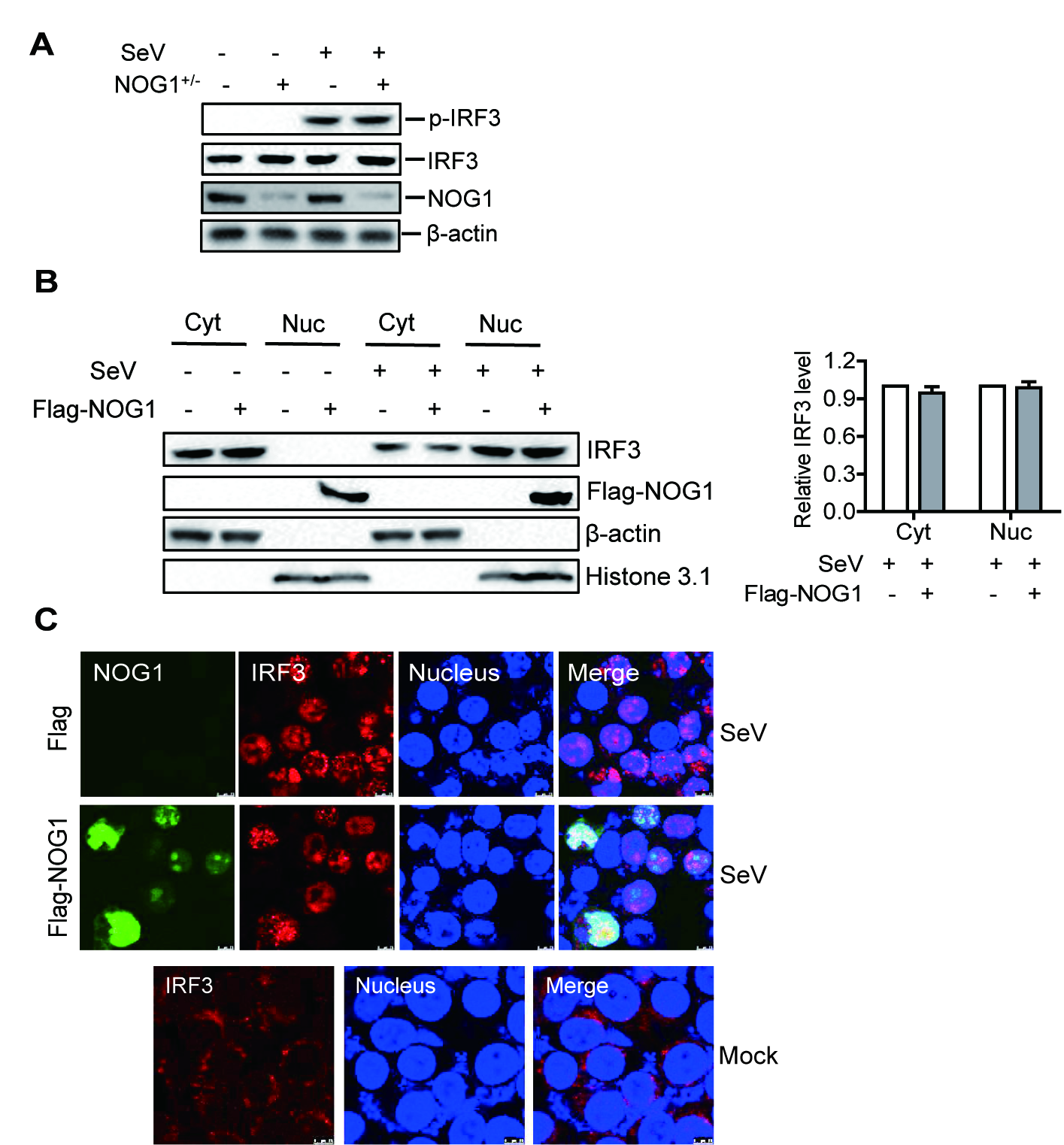

Supplement: S8 Fig — (A) WT and NOG1+/- cells were mock-infected or infected with SeV for 12 h, the cells were collected, and the levels of IRF3 phosphorylation were detected by Western blotting. (B) HEK-293T cells were transfected with 3 μg of Flag-NOG1-expressing plasmid. At 24 hpt, the cells were mock-infected or infected with SeV for 12 h. Cells were harvested and subjected to nuclear and cytoplasmic fractionation. The expression of IRF3 in the cytoplasm (Cyt) and nucleus (Nuc) was detected by Western blotting, respectively. Histone 3.1 was used as a nuclear loading control and marker, and β-actin was used as a cytosolic loading control and marker. The abundance of IRF3 was quantified using ImageJ software. (C) HEK-293T cells transfected with 0.5 μg of Flag empty vector or Flag-NOG1-expressing plasmid were mock-infected or infected with SeV for 12 h. The subcellular localization of NOG1 and IRF3 was detected by IFA. (TIF) [file ppat.1011511.s008.tif]

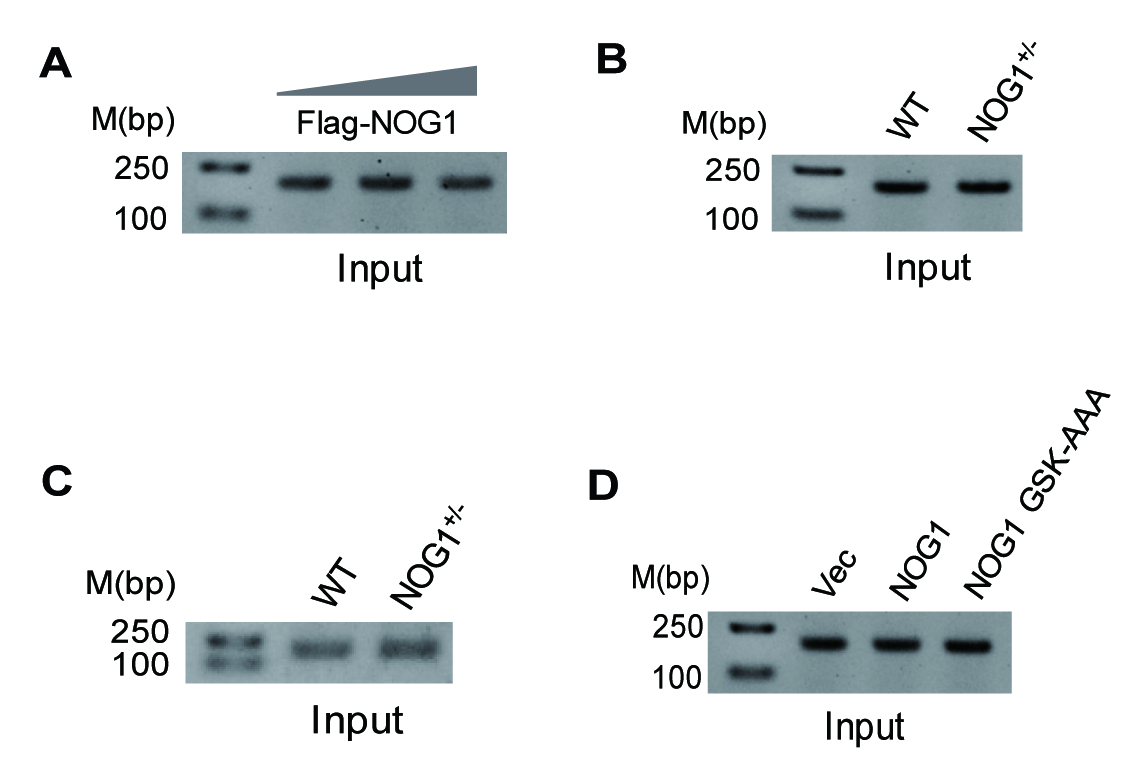

Supplement: S9 Fig — A, B, C, and D represent the input DNA levels of Figs 7A, 7B, 7C and 8C, respectively. (TIF) [file ppat.1011511.s009.tif]

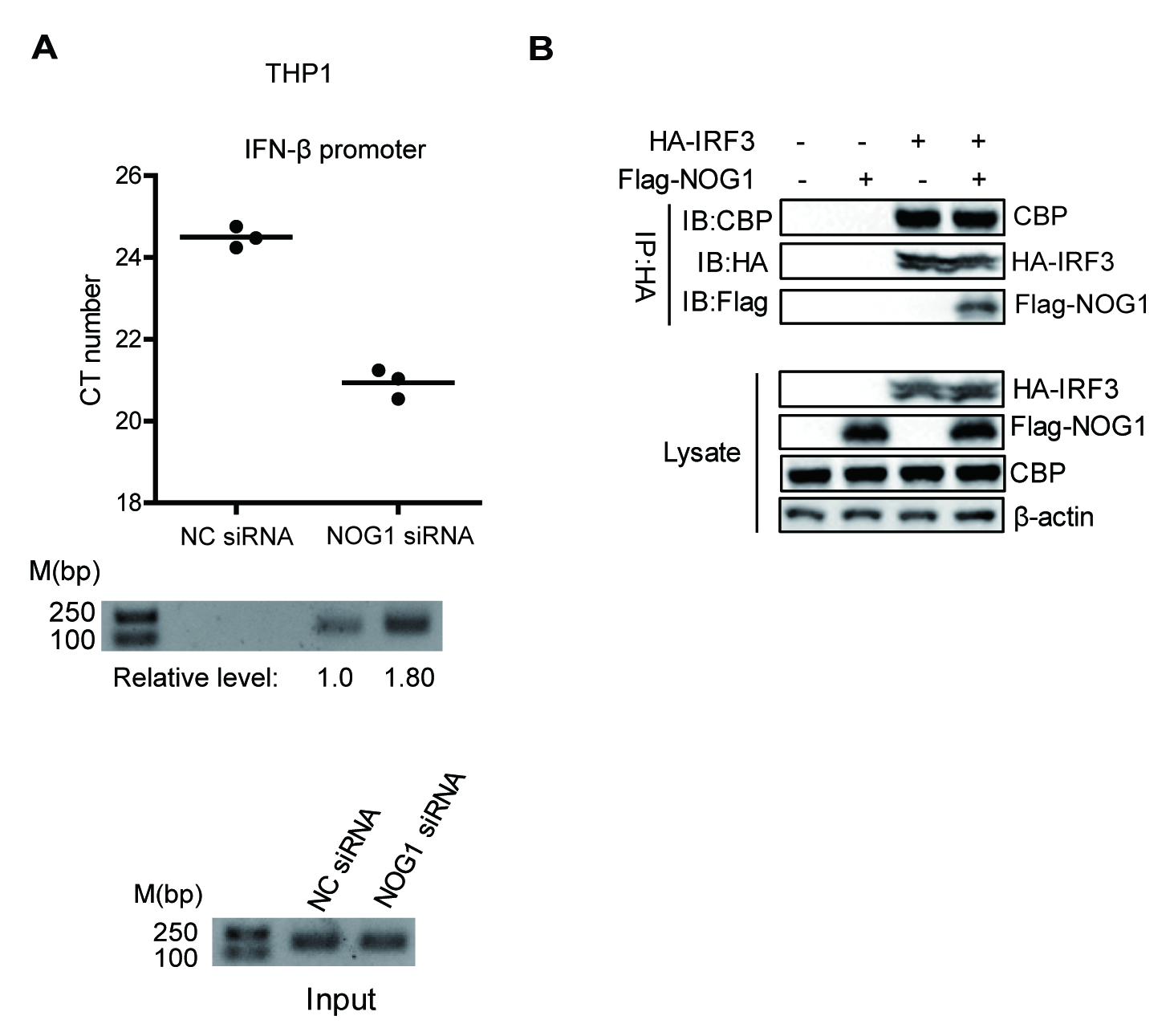

Supplement: S10 Fig — (A) THP1 cells transfected with 150 nM of NOG1 or NC siRNA were immunoprecipitated with anti-IRF3 antibody. The impact of NOG1 on IRF3 binding onto IFN-β promoter was analyzed by quantitative ChIP assay. (B) HEK-293T cells transfected with Flag-NOG1 and/or HA-IRF3 expression plasmids were infected with SeV for 12 h, and the cell lysates were immunoprecipitated with anti-HA antibody and subjected to Western blotting. (TIF) [file ppat.1011511.s010.tif]
